# Identification and Pathogenicity of Pestalotiod Fungi Associated with Woody Oil Plants in Sichuan Province, China

**DOI:** 10.3390/jof8111175

**Published:** 2022-11-08

**Authors:** Wen-Li Li, Asha J. Dissanayake, Tian Zhang, Sajeewa S. N. Maharachchikumbura, Jian-Kui Liu

**Affiliations:** School of Life Science and Technology, Center for Informational Biology, University of Electronic Science and Technology of China, Chengdu 611731, China

**Keywords:** 6 new taxa, *Neopestalotiopsis*, *Pestalotiopsis*, phylogeny, *Seiridium*, taxonomy

## Abstract

Pestalotiod fungi are associated with a wide variety of plants worldwide and occur as endophytes, pathogens, and saprobes. The present study provides an updated phylogeny for genera *Neopestalotiopsis*, *Pestalotiopsis,* and *Seiridium* using fresh collections from woody oil plants (*Camellia oleifera*, *Olea europaea*, *Paeonia suffruticosa*, *Sapium sebiferum,* and *Vernicia fordii*) in Sichuan Province, China. We coupled morphology and combined sequence data analyses of ITS, *tub2*, and *tef1-α* for *Neopestalotiopsis* and *Pestalotiopsis*, with ITS, LSU, *tub2*, *tef1-α*, and *rpb2* for *Seiridium*. Three novel species of *Neopestalotiopsis* (*N. mianyangensis*, *N. paeonia-suffruticosa*, *N. terricola*) and three of *Seiridium* (*S. guangyuanum*, *S. vernicola, S. oleae*), were found. Three other species, *Pestalotiopsis kenyana*, *Seiridium ceratosporum,* and *S. rosarum* were identified and reported as new records. All isolated species are fully described and illustrated. Additionally, the sexual morph of *Pestalotiopsis kenyana* is described for the first time. Pathogenicity tests revealed that *Neopestalotiopsis mianyangensis*, *N. paeonia-suffruticosa, N. terricola, Pestalotiopsis kenyana, Seiridium guangyuanum*, *S. vernicola*, and *S. oleae* are pathogenic on detached olive leaves.

## 1. Introduction

Woody oil plant is a general term for the fruits, leaves or seeds of woody plants that are used for oil extraction. It is a renewable resource for many oil products, such as edible oils, aromatic oils, industrial oils, and oils for bioenergy [1]. Woody oil plants have a long history of cultivation in China and are commonly grown in Sichuan, Yunnan, and Hunan Provinces [2]. About 8000 woody oil plant species are known in China, and over 300 of them contain above 20% fat content in fruits or seeds, such as *Camellia oleifera*, *Sapium sebiferum*, *Vernicia fordii*, etc. [3]. Statistical data from 2017 indicate that plantations of these plants produced 1,024,500 tons of oil annually (State-owned Forest Farms and Nurseries Station, State Forestry Administration of China, 2018).

The expanding cultivation of woody oil plants, especially *Camellia oleifera*, *Olea europaea*, *Paeonia suffruticosa*, and *Vernicia fordii* has attracted increasing attention of plant pathologists due to various fungal diseases. Several studies have reported the fungal pathogens causing diseases of woody oil plants, such as *Alternaria* [4], *Colletotrichum* [4,5], *Fusarium* [6,7], *Neopestalotiopsis*, and *Pestalotiopsis* [4,8].

Members of *Sporocadaceae* are known as pestalotiod fungi, comprising common phytopathogens that cause diseases, including shoot dieback, leaf spots, and fruit rots on many different woody plants worldwide [9,10,11,12,13]. For example, *Seiridium* spp. (*S. cardinale*, *S. cupressi*, *S. unicorne*) cause cypress canker disease; *Neopestalotiopsis* spp. (*N. camelliae-oleiferae*, *N. clavispora*, etc.) and *Pestalotiopsis* spp. (*Pestalotiopsis camellia*, *P. furcate*, *P. longiseta*, *P. menhaiensis*, *P. sichuanensis*, etc.) are pathogenic for tea gray blight disease [4,14,15,16,17]. Most species of *Sporocadaceae* have multi-septate and fusiform conidia with appendages at one or both ends, frequently with some melanized cells. Jaklitsch et al. [18] accepted several pestalotiod genera (e.g., *Bartalinia*, *Discosia*, *Pestalotiopsis*, *Seimatosporium*, *Seiridium*, *Truncatella*) within *Sporocadaceae*. Subsequently, Liu et al. [19] accepted 30 genera in the family. The genus *Seiridium* was established by Nees with *S. marginatum* as the type [20] and it can be easily distinguished from other genera in *Sporocadaceae* by having 5-septate conidia. Bonthond et al. [21] recommended the use of four loci (ITS, *tef1-α*, *tub2*, and *rpb2*) to resolve the phylogenetic relationship of *Seiridium*. Maharachchikumbura et al. segregated *Neopestalotiopsis* from *Pestalotiopsis* based on conidial pigmentation, conidiophores, and multi-locus phylogenetic analyses [13]. Maharachchikumbura et al. [10] proved that multi-locus phylogenetic analysis (ITS, *tef1-α* and *tub2*) is reliable in addressing the challenges of identification of *Neopestalotiopsis* and *Pestalotiopsis*. The *tef1-α* gene, in particular, is considered a good molecular marker to separate most pestalotiod species. This conclusion was followed by subsequent studies [4,22,23,24].

To provide a stable molecular-based phylogeny to this morphologically highly similar fungal group, we have been studying pestalotiod taxa from woody oil plants in Sichuan Province, China. The study aimed to resolve pestalotiod taxa at the species level based on both morphological characteristics and multi-locus phylogenetic analyses while also testing the pathogenicity of these fungi on detached olive leaves.

## 2. Materials and Methods

### 2.1. Specimen Collection, Examination, and Fungal Isolation

**Figure 1:**
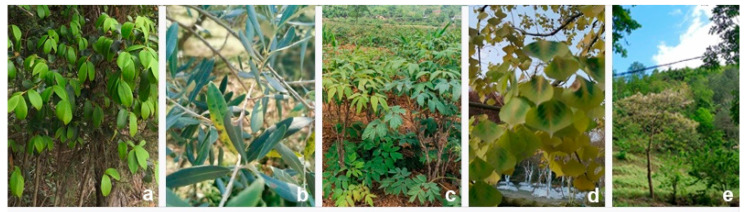
Hosts of pestalotiod species collected in this study (**a**) *Camellia oleifera*, (**b**) *Olea europaea*, (**c**) *Paeonia suffruticosa*, (**d**) *Sapium sebiferum*, (**e**) *Vernicia fordii*.

Diseased branches and leaves of woody oil plants (*Camellia oleifera*, *Olea europaea*, *Paeonia suffruticosa*, *Sapium sebiferum*, and *Vernicia fordii*, Figure 1) were collected from Sichuan Province, China (detailed information of the collection sites are mentioned in Section 3.2). Specimens were taken to the laboratory and observed using a Motic SMZ 168 stereo microscope. Micro-morphological characteristics were observed and photographed with a Nikon ECLIPSE Ni compound microscope fitted with a Canon EOS 600D digital camera. Measurements were taken by Tarosoft Image Frame Work program v. 0.9.7 and the images for figures were processed with Adobe Photoshop CS6 Extended v. 13.0 software. Fungal isolates were obtained by the single spore isolation method. Germinated conidia were transferred aseptically to potato dextrose agar (PDA) plates and grown at 25 °C in daylight. Colony color [25] and other characters were observed and measured after two weeks.

The isolates obtained in this study were deposited at the China General Microbiological Culture Collection Center (CGMCC), in Beijing, China and the University of Electronic Science and Technology Culture Collection (UESTCC), in Chengdu, China. Specimens were deposited at the Herbarium of Cryptogams, Kunming Institute of Botany Academia Sinica (KUN-HKAS), in Kunming, China and at the Herbarium of the University of Electronic Science and Technology (HUEST), in Chengdu, China. New taxa were established based on recommendations outlined by Jeewon and Hyde [26]. The scientific names of the new taxa were registered in MycoBank (www.mycobank.com accessed on 25 August 2022)) and Facesoffungi [27].

### 2.2. DNA Extraction, PCR Amplification, and Sequencing

Fungal genomic DNA were extracted from fresh mycelia scraped from the margin of colonies on PDA that had been incubated at 25 °C for 14 d, following the manufacturer’s instructions of the EZ geneTM fungal gDNA kit (GD2416). Fungal genomic DNA were obtained directly from disinfected fruiting bodies for the species that did not germinate. Polymerase chain reaction (PCR) was conducted for five genes of the new collections: internal transcribed spacer (ITS: ITS1-5.8S-ITS2), the large subunit of the nuclear ribosomal RNA genes (LSU), partial β-tubulin (*tub2*), translation elongation factor-1 alpha (*tef1-α*), and RNA polymerase II core subunit (*rpb2*) gene. The primers used were ITS5/ITS4 for ITS [28], LR0R/LR5 for LSU [29], T1/Bt2b for *tub2* [30,31], EF1-728F/EF2 for *tef1-α* [32,33], and fRPB2-5f/fRPB2-7cR for *rbp2* [34,35]. The amplification reactions were performed in 25 μL of PCR mixtures containing 9.5 μL ddH_2_O, 12.5 μL 2 × PCR MasterMix (Kangwei Co., Guangzhou, China), 1 μL DNA template, and 1 μL of each primer. The PCR thermal cycle program for ITS, LSU, *tub2*, *tef1-α*, and *rpb2* amplification was as follows: an initial denaturation step of 5 min at 94 °C followed by 35 cycles of 30 s at 94 °C, 50 s at 52 °C (ITS, LSU, *tub2*, *tef1-α*) or 55 °C (*rpb2*) and 1 min at 72 °C, and a final elongation step of 7 min at 72 °C. The sequences obtained in this study were supplemented with additional sequences from GenBank (Table 1; Table S1).

### 2.3. Sequence Alignment and Phylogenetic Analyses

A concatenated dataset of the ITS, LSU, *tub2*, *tef1-α*, and *rpb2* sequences were used for phylogenetic analyses of the genus *Seiridium*; ITS, *tef1-α*, and *tub2* for *Neopestalotiopsis* and *Pestalotiopsis*. The dataset of *Neopestalotiopsis* consisted of 112 isolates (*Pestalotiopsis diversiseta* MFLUCC 12-0287 as outgroup taxon), 139 isolates for *Pestalotiopsis* (*Neopestalotiopsis magna* MFLUCC 12-0652 as outgroup taxon) and 52 isolates for *Seiridium* (*Neopestalotiopsis protearum* CBS 114,178 as outgroup taxon). Sequences were aligned for each gene separately using the MAFFT v.7.110 online program (http://mafft.cbrc.jp/alignment/server/ (accessed on 15 May 2022)) [36] and manually optimized using BioEdit v.7.0.9 [37]. Maximum likelihood (ML), maximum parsimony (MP), and Bayesian analyses were carried out following Dissanayake et al. [38]. The evolutionary models of nucleotide substitution were selected independently for each locus using MrModeltest 2.2. The phylogenetic tree was visualized by FigTree v.1.4.2 [39].

### 2.4. PHI Analysis

PHI analysis (Pairwise Homoplasy Index) is used for confirming new species with low statistical support and significant tree lengths. The evidence of significant genetic recombination between the novel species and its closely related species was conducted (If Fw > 0.05).

### 2.5. Pathogenicity Tests on Olive Leaves

Olive (*Olea europaea*), widely cultivated as a woody oil plant in Sichuan Province, was selected as the material for the pathogenicity tests. Fresh and healthy leaves were collected from three-year-old olive trees in Chengdu olive cultivation base. Two experimental methods were established to check the infection ability and the virulence of the pestalotiod-like fungi on olive leaves. The olive leaves were washed thoroughly in running water and surface disinfected for 1 min in 75% alcohol, washed with sterilized double distilled water, and dried on sterile filter papers.

Unwounded method: three olive leaves were inoculated per fungal species. A mycelial plug (5 mm diam.), taken from the edge of a two weeks-old growing culture, was placed on the olive leaf. Another two olive leaves that served as the negative control were each inoculated with a sterile PDA plug. Wounded method: an artificial injury was made using a sterilized dissecting needle on the olive leaf surface and a 5 mm diam. A mycelial plug was placed over the wound. All leaves were placed in a humid chamber at 25 °C for 7 d.

Development of any disease symptoms were checked daily following inoculation, and the lesion length was measured after 7 d using a digital caliper. After 14 d, re-isolation was conducted from the margin of the necrotic tissue to recover the infected fungi and to meet Koch’s postulates. Additionally, isolation from the negative controls was conducted to verify that no endophytic pestalotiod fungus was present.

### 2.6. Statistical Analysis

Data related to the pathogenicity test were analyzed with SPSS version 24 software (SPSS Inc., Chicago, IL, USA) by one-way variance analysis. The means were compared using Duncan’s test at a significance level of *p* ≤ 0.05.

## 3. Results

### 3.1. Phylogenetic Analyses

**Figure 2:**
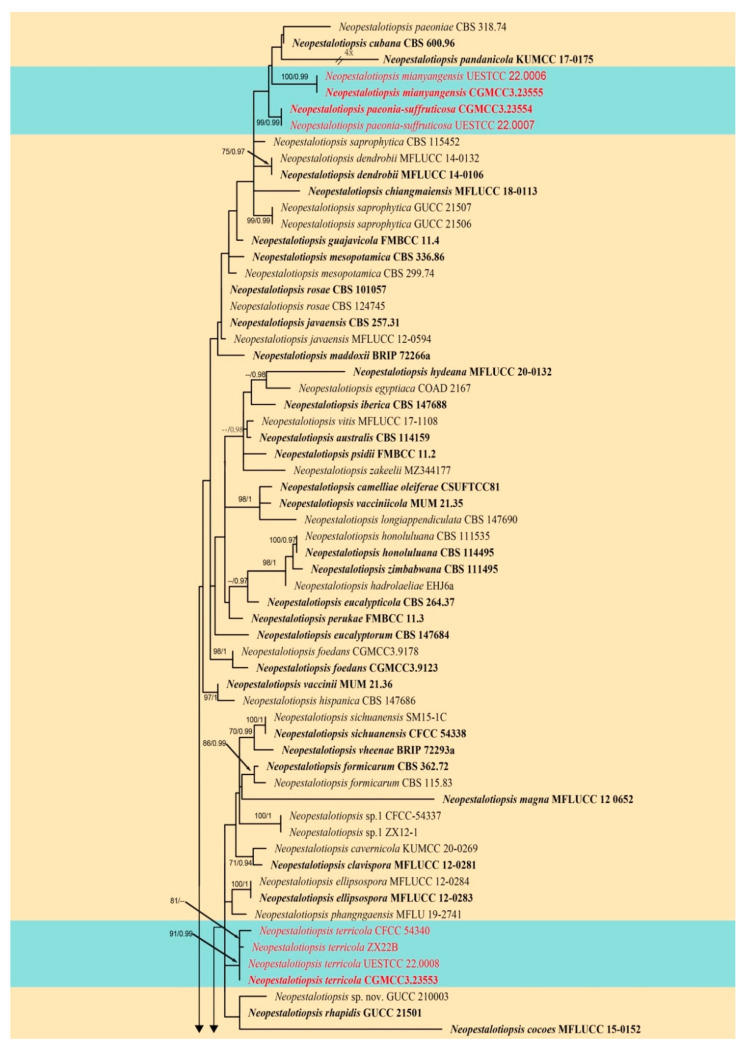

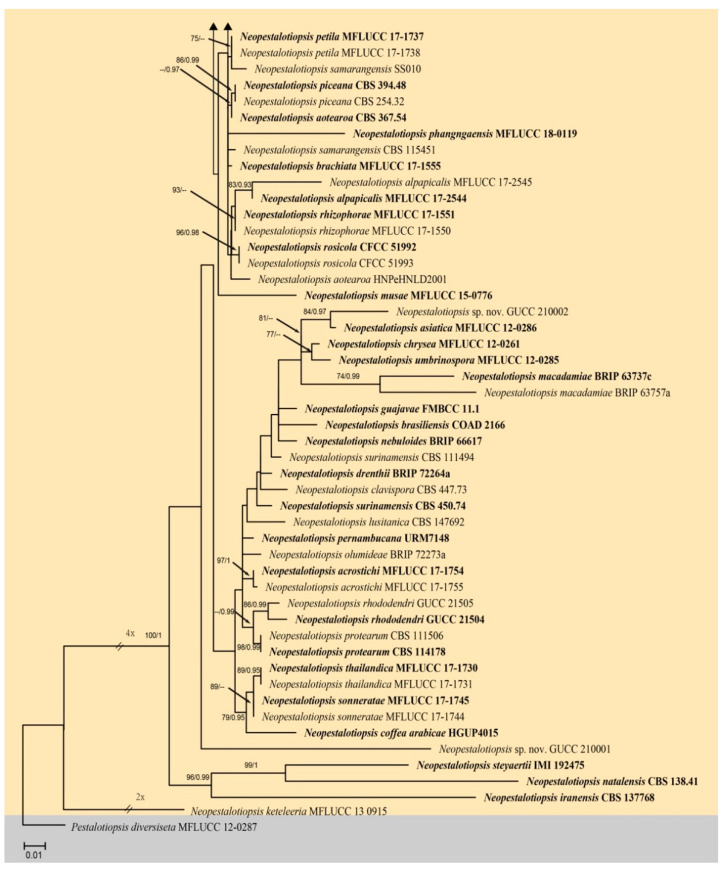
Phylogram generated from RAxML analysis based on combined ITS, *tef1-α*, and *tub2* sequence data of *Neopestalotiopsis* isolates. The tree was rooted to *Pestalotiopsis diversiseta* (MFLUCC 12-0287). The ML, MP bootstrap supports (≥60%) and BI posterior probabilities (≥0.90 PP) supports are given near the nodes, respectively. Isolates from this study are marked in red and ex-type strains are marked in bold.

The first combined ITS, *tub2*, and *tef1-α* sequence dataset was analyzed to infer the interspecific relationships within *Neopestalotiopsis*. The concatenated data matrix consisted of sequences of 112 isolates, including the outgroup taxon *Pestalotiopsis diversiseta* (MFLUCC 12-0287). A total of 1826 characters including gaps (502 for ITS, 753 for *tub2*, and 571 for *tef1-α*) were included in the alignment. For the Bayesian inference, the HKY + I + G model with invgamma-distributed rate was selected for ITS, GTR + I + G model with invgamma-distributed rate was selected for *tub2* and the HKY + G model with gamma-distributed rate was selected for *tef1-α.* The maximum likelihood tree confirmed a similar tree topology to the Bayesian consensus tree, and the best-scoring ML tree is shown in Figure 2. The novel species *Neopestalotiopsis mianyangensis* (UESTCC 22.0006) clusters closest to *N. cubana* (CBS 600.96), *N. paeoniae* (CBS 318.74), and *N. pandanicola* (KUMCC 17-0175). *Neopestalotiopsis paeonia-suffruticosa* forms a separate branch, and it is phylogenetically related to the above four taxa, while our new strains *Neopestalotiopsis terricola* (CGMCC3.23553, UESTCC 22.0033) group together with previously reported species *Neopestalotiopsis* sp. (CFCC 54340) and *Neopestalotiopsis* sp. (ZX22B) in a monophyletic clade.

**Figure 3:**
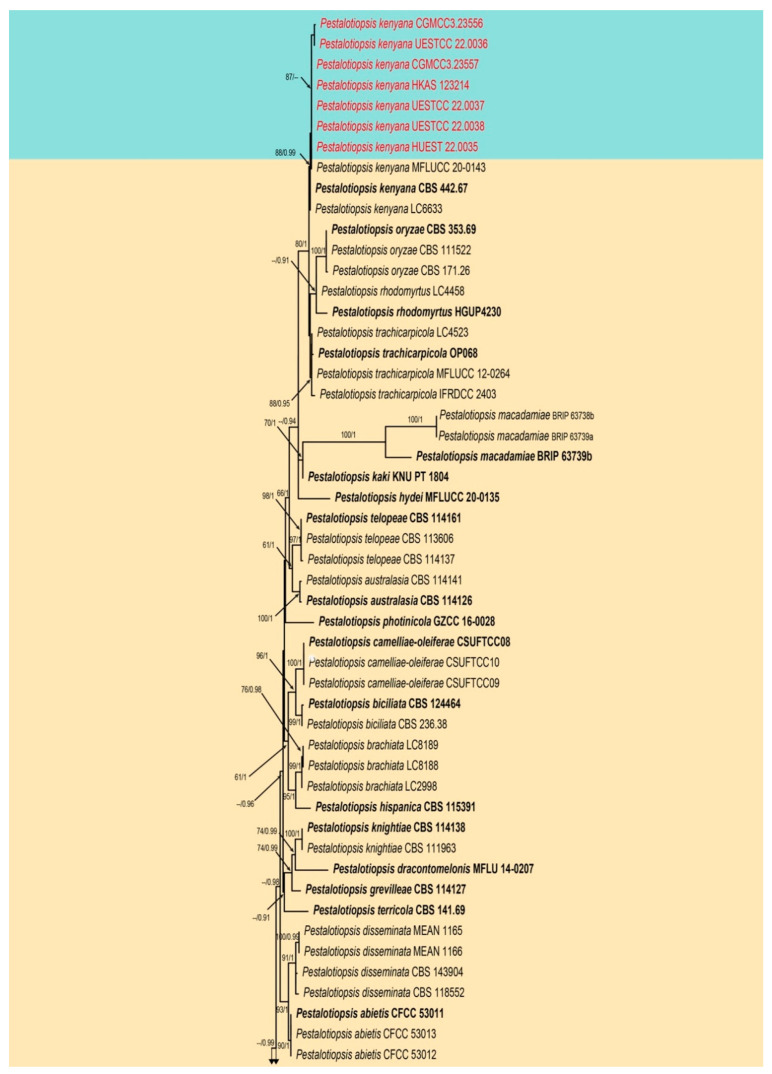

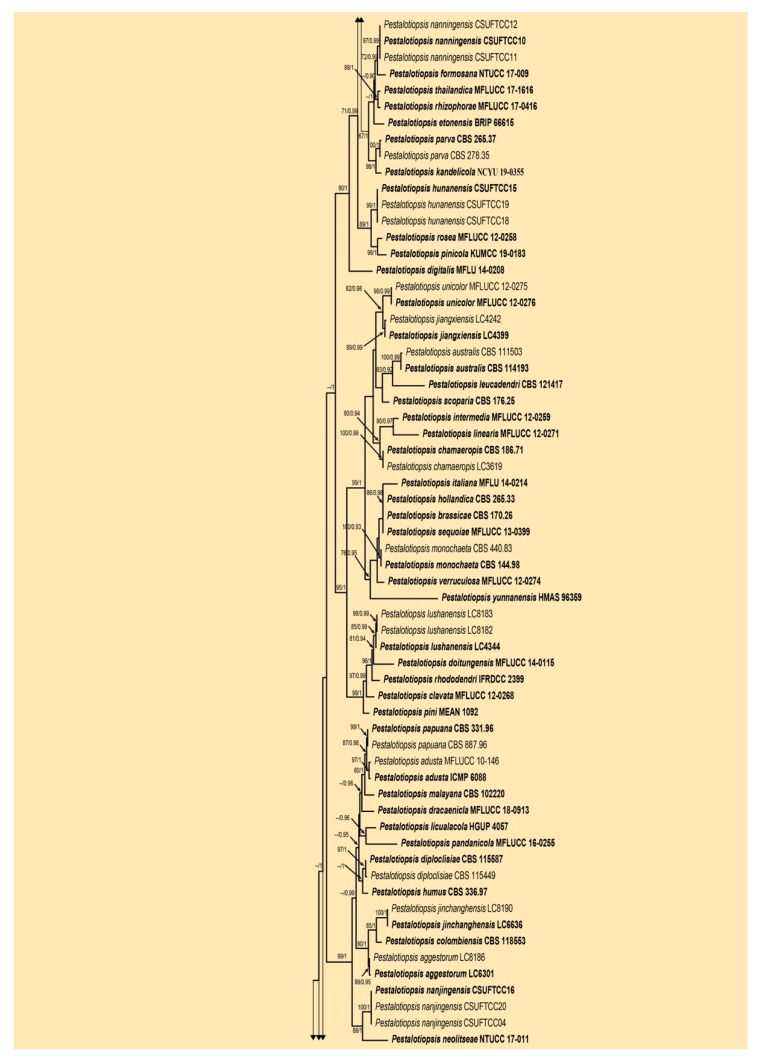

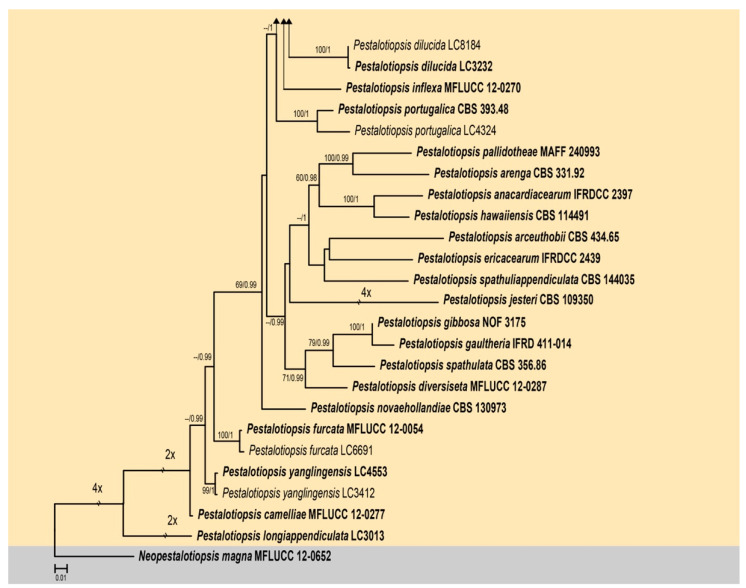
Phylogram generated from RAxML analysis based on combined ITS, *tef1-α*, and *tub2* sequence data of *Pestalotiopsis* isolates. The tree was rooted to *Neopestalotiopsis magna* (MFLUCC 12-0652). The ML, MP bootstrap supports (≥60%) and BI posterior probabilities (≥0.90 PP) supports are given near the nodes, respectively. Isolates from this study are marked in red and ex-type strains are marked in bold.

The second combined ITS, *tub2*, and *tef1-α* sequence dataset comprised sequences of 139 isolates of *Pestalotiopsis* including *Neopestalotiopsis magna* (MFLUCC 12-0652) as the outgroup taxon. A total of 1958 characters including gaps (548 for ITS, 823 for *tub2*, and 587 for *tef1-α*) were included in the alignment. For the Bayesian inference, the HKY + I + G model with invgamma-distributed rate was selected for ITS, the GTR + I + G model with invgamma-distributed rate was selected for *tub2*, and the HKY + G model with gamma-distributed rate was selected for *tef1-α*. The maximum likelihood tree confirmed the similar tree topology to the Bayesian consensus tree, and the best-scoring ML tree is shown in Figure 3. Seven strains obtained from this study clustered with *Pestalotiopsis kenyana* (CBS 442.67, LC6633) with moderate bootstrap support (ML 88%, BS 0.99).

**Figure 4:**
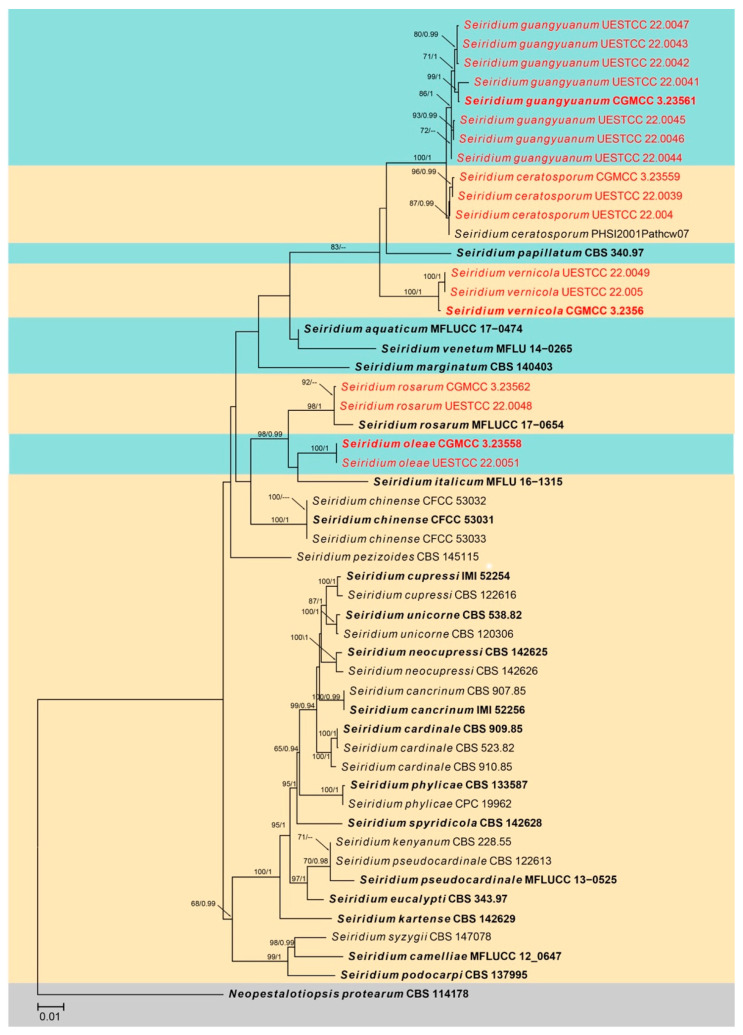
Phylogram generated from RAxML analysis based on combined LSU, ITS, *tub2*, *tef1-α* and *rpb2* sequence data of *Seiridium* isolates. The tree was rooted to *Neopestalotiopsis protearum* (CBS 114178). The ML, MP bootstrap supports (≥60%) and BI posterior probabilities (≥0.90 PP) supports are given near the nodes, respectively. Isolates from this study are marked in red and ex-type strains are marked in bold.

The third combined LSU, ITS, *tub2*, *tef1-αi*, and *rpb2* sequence dataset comprised sequences of 52 isolates of *Seiridium* with *Neopestalotiopsis protearum* (CBS 114178) as the outgroup taxon. A total of 3886 characters including gaps (851 for LSU, 588 for ITS, 780 for *tub2*, 617 for *tef1-α*, and 1050 for *rpb2*) were included in the phylogenetic analysis. For the Bayesian inference, the HKY + I + G model with invgamma-distributed rate was selected for ITS and *tub2*, the HKY + I model with propinv-distributed rate was selected for LSU, the SYM + I + G model with invgamma-distributed rate was selected for *rpb2*, and the HKY + G model with gamma-distributed rate was selected for *tef1-α*. The maximum likelihood tree confirmed the similar tree topology to the Bayesian consensus tree, and the best-scoring ML tree is shown in Figure 4. Eighteen strains from this study clustered into five distinct clades in *Seiridium*, representing three new species and two known species.

### 3.2. Taxonomy

#### 3.2.1. *Neopestalotiopsis mianyangensis* W.L. Li and Jian K. Liu sp. nov. (Figure 5)

MycoBank: MB 845406; Facesoffungi number: FoF 12746

Etymology: The name reflects the location, Mianyang city, where the fungus was collected.

Holotype: HKAS 123211

*Pathogenic* on diseased branches of *Paeonia suffruticosa*. Sexual morph: not observed. Asexual morph: *Conidiomata* 139–143 μm high, 245–250 μm diam. ($\bar{x}$ = 141 × 248 μm, *n* = 15), globose, solitary, semi-immersed, black, exuding dark brown to black masses of conidia. The *Conidiomata wall* is 24–30 μm thick at the sides, not well defined, comprising brown, thin-walled cells of textura angularis, with lighter cells at the base fusing into the host tissue. *Conidiophores* are indistinct, often reduced to conidiogenous cells. *Conidiogenous cells* 3–5 × 2.1–2.5 μm ($\bar{x}$ = 4 × 2.5 μm, *n* = 30), mostly integrated, ampulliform to lageniform, hyaline to light brown, smooth-walled, single, with truncated apex. *Conidia* 19–23 × 5.5–7 μm ($\bar{x}$ = 21 × 6.5 μm, *n* = 30), fusoid, ellipsoid, straight to slightly curved, 4-septate; conical to obconical basal cell with a truncated base, hyaline, rough and thin-walled, 3–4 μm long, often with a small basal appendix; three medial cells with light brown to dark pigmentation with a rough wall, and one septum darker than the rest of the cell (second cell from the base pale brown, 4–5 μm long; third cell golden brown 4–5.5 μm long; fourth cell brown, 4–5.5 μm long); apical cell 3–4 μm long, hyaline, cylindrical, thin and smooth-walled with three apical tubular appendages, not arising from the apical crest, but each inserted at a different locus in the upper half of the apical cell, unbranched, filiform, 5.5–11 μm; single basal appendix, tubular, unbranched, centric, 3–4 μm long, mean conidium length/width ratio = 3.3:1.

**Figure 5:**
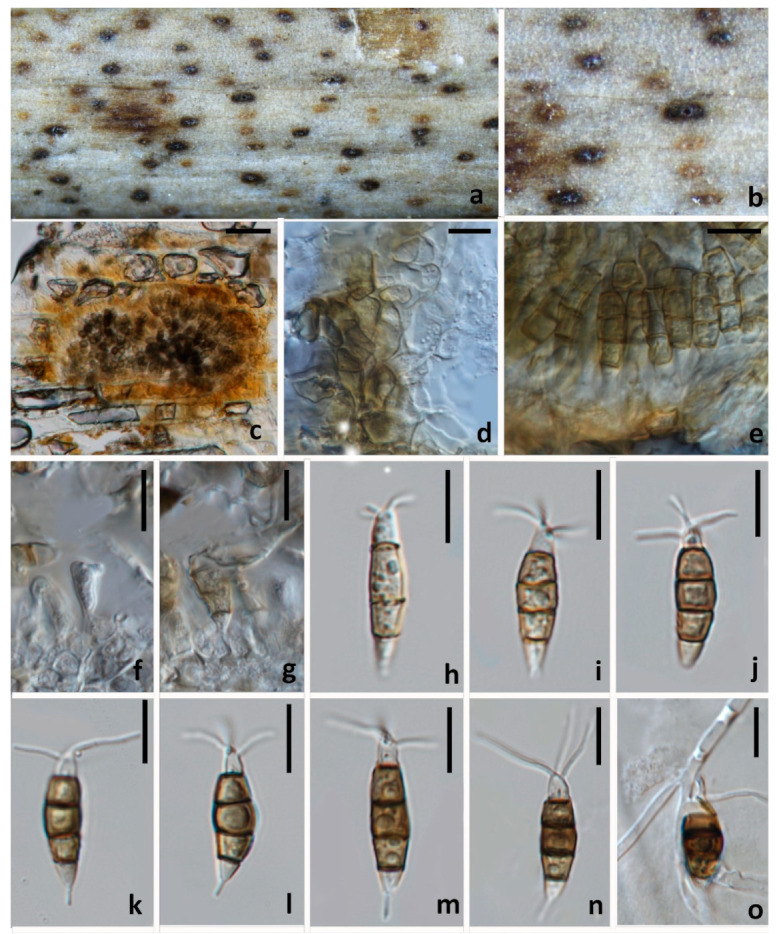
*Neopestalotiopsis mianyangensis* (HKAS 123211, holotype) (**a**,**b**) Appearance of conidiomata on the host. (**c**) Transverse section of conidioma. (**d**) Conidiomatal wall. (**e**) Sporodochium. (**f**,**g**) Conidiogenous cells and conidia. (**h**–**n**) conidia. (**o**) Germinated conidium. Scale bars: (**c**) = 50 μm, (**d**–**o**) = 10 μm.

Culture characteristics: Colonies on PDA reaching 70 mm diam. after 7 d at 25 °C, filamentous to circular, medium dense, with white sparse mycelium.

Material examined: CHINA, Sichuan Province, Mianyang city, on a diseased branch of *Paeonia suffruticosa*, 10 September 2021, W.L. Li, YMD 451 (HKAS123211, holotype), ex-type living culture, CGMCC3.23554; *ibid*., HUEST 22.0066, isotype, ex-isotype living culture, UESTCC 22.0066.

**Figure 6:**
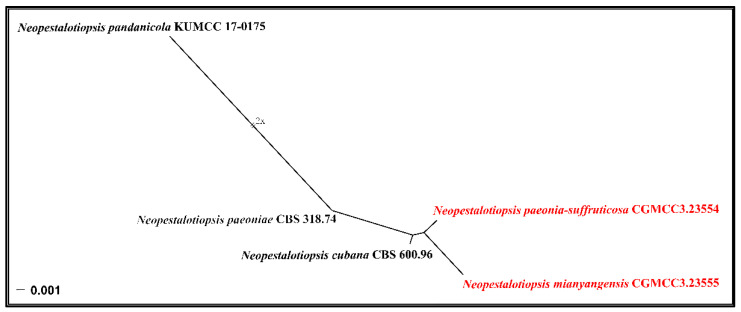
Split graphs showing the results of PHI test of *Neopestalotiopsis mianyangensis* and *N. paeonia-suffruticosa* with their most closely related species (Fw = 0.2781). The new taxa are shown in red.

Notes: *Neopestalotiopsis mianyangensis* (UESTCC 22.0006) forms a sister clade to *N. cubana* (CBS 600.96), *N. pandanicola* (KUMCC 17–0175), and *N. paeoniae* (CBS 318.74) in a poorly-supported clade (Figure 2). *Neopestalotiopsis mianyangensis* differs from *N. cubana* by its shorter conidiogenous cells (3–5 μm vs. 5–12 μm) and shorter apical appendages (5.5–11 μm vs. 21–27 μm), as well as narrower conidia (5.5–7 μm vs. 8–9.5 μm). *Neopestalotiopsis pandanicola* can be morphologically distinguished from *N. mianyangensis* by larger conidia (27–35 μm vs. 19–23 μm) and longer appendages (9.5–26 μm vs. 5.5–11 μm). *Neopestalotiopsis paeoniae* was originally described from *Anacardium occidentale* [19]. Unfortunately, no living culture was obtained for this species, and only ITS and *tef1-α* sequences are available. *Neopestalotiopsis mianyangensis* and *N. paeoniae* had seven base pairs differences in *tef1-α* loci without gaps. The PHI analysis further confirms that *N. mianyangensis* has no significant genetic recombination with closely related species (Fw > 0.05, Figure 6). We thus introduced *N. mianyangensis* as a new species.

#### 3.2.2. *Neopestalotiopsis paeonia-suffruticosa* W.L. Li and Jian K. Liu., sp. nov**.** (Figure 7)

MycoBank: MB 845407; Facesoffungi number: FoF 12747

Etymology: The name reflects the host plant Paeonia suffruticosa from which it was isolated.

Holotype: HKAS 123212

*Pathogenic* on diseased branches of *Paeonia suffruticosa*. Sexual morph: not observed. Asexual morph: *Conidiomata* 88–92 μm high, 218–229 μm diam. ($\bar{x}$ = 90 × 223, *n* = 15), globose, solitary, semi-immersed, black, exuding dark brown to black masses of conidia. The *Conidiomata wall* is 16–19 μm thick at the sides, not well defined, comprising brown, thin-walled cells of *textura angularis*, with lighter cells at the base fusing into the host tissue. *Conidiophores* are indistinct, often reduced to conidiogenous cells. *Conidiogenous cells* 5–6.5 × 2.5–3 μm ($\bar{x}$ = 5.5 × 2.5 μm, *n* = 30), mostly integrated, ampulliform to lageniform, hyaline to light brown, smooth-walled, single, with truncated apex. *Conidia* 20–23 × 9–11 μm ($\bar{x}$ = 21 × 9.5 μm, *n* = 30), fusoid, ellipsoid, straight to slightly curved, 4-septate; conical to obconical basal cell with a truncated base, hyaline, rough and thin-walled, 3–4 μm long, often with a small basal appendix; three medial cells with light brown to dark pigmentation with a rough wall, and one septum darker than the rest of the cell (second cell from the base pale brown, 4–5.5 μm long; third cell golden brown 5.5–7 μm long; fourth cell brown, 4.5–6 μm long); apical cell of 2.5–3.5 μm long, hyaline, cylindrical, thin and smooth-walled with three apical tubular appendages, not arising from the apical crest, but each inserted at a different locus in the upper half of the apical cell, unbranched, filiform, flexuous, 22.5–34 μm long; single basal appendix, tubular, unbranched, centric, 3.5–7.5 μm long, mean conidium length/width ratio = 2.2:1.

**Figure 7:**
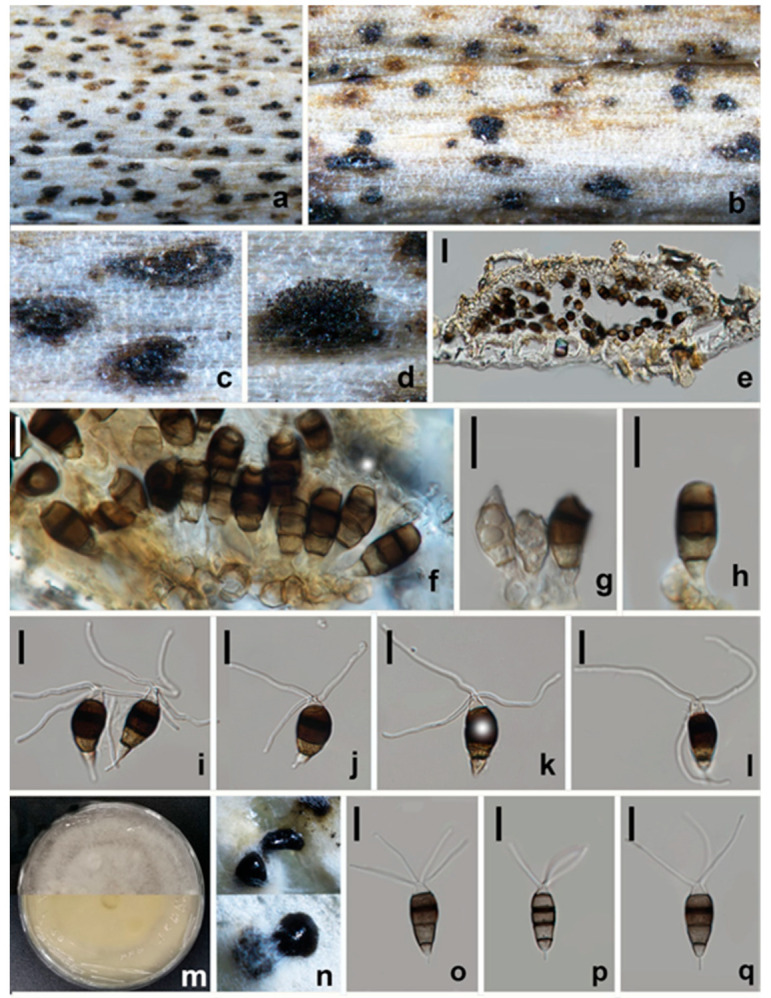
*Neopestalotiopsis paeonia-suffruticosa* (HKAS 123212, holotype) (**a**–**d**) Appearance of conidiomata on the host. (**e**) Vertical section of conidioma. (**f**) Sporodochium. (**g**,**h**) Conidiogenous cells and conidia. (**i**–**l**,**o**–**q**) Conidia. (**m**) Upper and reverse view of colony on PDA. (**n**) Appearance of conidia mass on PDA. Scale bars: (**e**) = 20 μm, (**f**–**l**,**o**–**q**) = 10 μm.

Culture characteristics: Colonies on PDA reaching 70 mm diam. after 7 d at 25 °C. Colonies filamentous to circular, medium dense, with white sparse mycelium, fruiting bodies black.

Material examined: CHINA, Sichuan Province, Mianyang city, on a diseased branch of *Paeonia suffruticosa*, 10 September 2021, W.L. Li, YMD 342a (HKAS 123212, holotype), ex-type living culture, CGMCC3.23554; *ibid*., HUEST 22.0033, isotype, ex-isotype living culture, UESTCC 22.0033.

Notes: Phylogenetically, *Neopestalotiopsis paeonia-suffruticosa* formed a distinct clade in the multi-locus tree and is sister to a poorly-supported clade containing *N. cubana* (CBS 600.96), *N. mianyangensis* (UESTCC 22.0006) and *N. pandanicola* (KUMCC 17–0175) (Figure 2). *Neopestalotiopsis paeonia-suffruticosa* differs morphologically from *N. cubana* by longer conidiogenous cells (up to 12 μm vs. 5–6.5 μm) and longer apical appendages (22.5–33.5 μm vs. up to 27 μm). Moreover, they also differed in six base pairs in *tef1-α* and one base pair in *tub2* loci without gaps. *Neopestalotiopsis pandanicola* differs from *N. paeonia-suffruticosa* in having larger conidia (27–35 μm vs. 20–23 μm μm) with 2(–3) apical tubular appendages. *Neopestalotiopsis mianyangensis* morphologically differs from *N. paeonia-suffruticosa* in having wider conidia (9–10 μm vs. 5.5–7) and longer appendages (apical appendages 22.5–34 μm vs. 5.5–11 μm, basal appendages 3.5–7.5 μm vs. 3–4 μm) (Table 2). As confirmed by PHI analysis (Fw > 0.05, Figure 6), no significant genetic recombination was observed with closely related species. Hence, we introduce *N. paeonia-suffruticosa* as a new species.

#### 3.2.3. *Neopestalotiopsis terricola* W.L. Li and Jian K. Liu, sp. nov. (Figure 8 and Figure 9)

MycoBank: MB 845408; Facesoffungi number: FoF 12748

Etymology: Named referring to the terrestrial habitat of this fungus.

Holotype: HKAS 123213

*Pathogenic* on diseased branches of *Paeonia suffruticosa*. Sexual morph: not observed. Asexual morph: *Conidiomata* globose, solitary, semi-immersed, black, excluding dark brown to black masses of conidia. *Conidiophores* indistinct, often reduced to conidiogenous cells. The *Conidiogenous cells* are 2.5–3.5 × 2–3 μm ($\bar{x}$ = 3 × 2.5 μm, *n* = 30), mostly integrated, ampulliform to lageniform, hyaline, smooth-walled, single, with truncated apex. *Conidia* 20–23 × 8–9.5 μm ($\bar{x}$ = 21.5 × 8.5 μm, *n* = 30), fusoid, ellipsoid, straight to slightly curved, 4-septate; conical to obconical basal cell with a truncated base, hyaline, rough, and thin-walled, 3–4 μm long, often with a small basal appendix; three medial cells with light brown to dark pigmentation with a rough wall (second cell from base pale brown, 4–5 μm long; third cell golden brown 5–6 μm long; fourth cell brown, 4.5–5 μm long); apical cell 2.5–4 μm long, hyaline, cylindrical, thin and smooth-walled with three apical tubular appendages, not arising from the apical crest, but each inserted at a different locus in the upper half of the apical cell, unbranched, filiform, 15–23 μm long; single basal appendix, tubular, unbranched, centric, 4–6 μm, mean conidium length/width ratio = 2.5:1.

**Figure 8:**
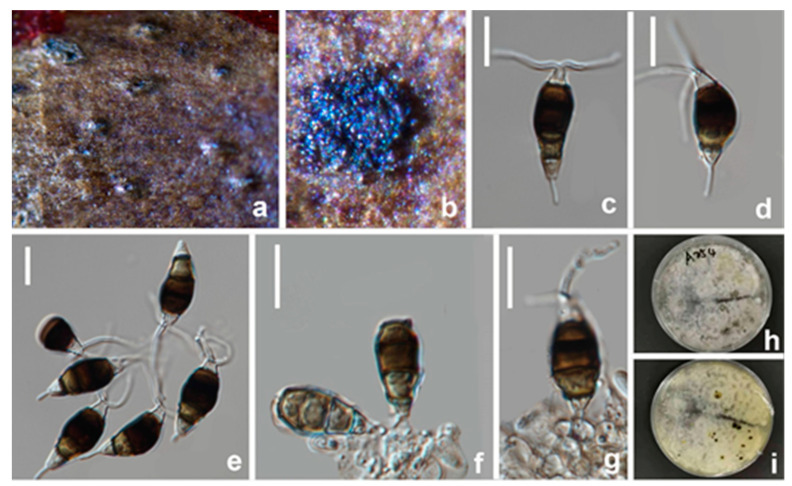
*Neopestalotiopsis terricola* (HKAS 123213, holotype) (**a**,**b**) Appearance of conidiomata on the host. (**c**–**e**) Conidia. (**f**,**g**) Conidiogenous cells and conidia. (**h**,**i**) Upper and reverse view of the colony on PDA. Scale bars: (**c**–**g**) = 10 μm.

**Figure 9:**
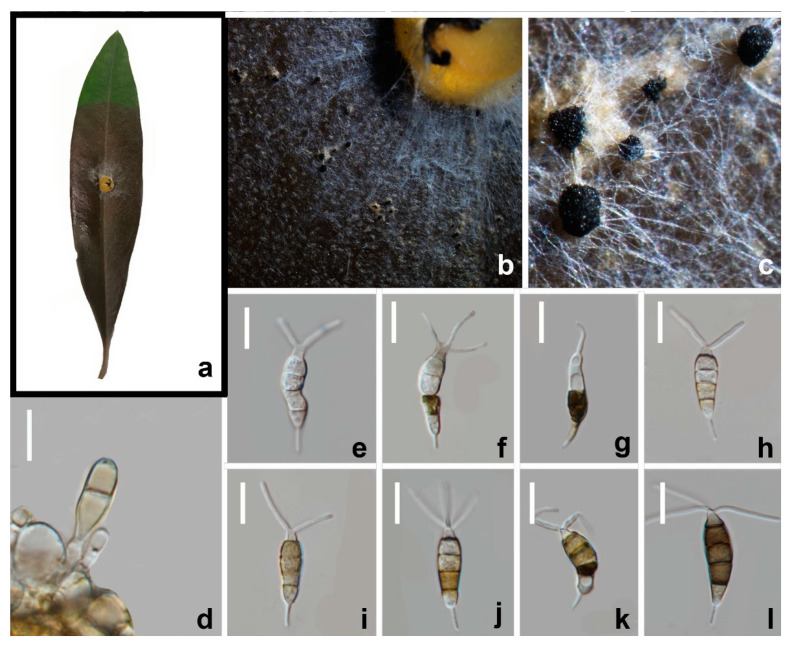
*Neopestalotiopsis terricola* (HKAS 123213) (**a**). *Olea europaea* leaves infected by *N. terricola* (**b**,**c**) Conidiomata on infected leaves. (**d**) Conidiogenous cells and conidia. (**e**–**l**) Conidia. Scale bars: (**d**–**l**) = 10 μm.

Culture characteristics: Colonies on PDA reaching 70 mm diam. After 7 d at 25 °C, filamentous to circular, medium dense, with white sparse mycelium, fruiting bodies black.

Material examined: CHINA, Sichuan Province, Mianyang city, on a diseased branch of *Paeonia suffruticosa*, 10 June 2021, W.L. Li, YMD A254 (HKAS123213, holotype), ex-type living culture, CGMCC3.23553; *ibid*., Chengdu city, on diseased leaf of *Olea europaea*, YMD A254b (HUEST 22.0034), living culture, UESTCC 22.0034.

Notes: Two strains (CGMCC3.23553, UESTCC 22.0034) obtained in this study clustered together with *Neopestalotiopsis* sp. (CFCC 54340) and *Neopestalotiopsis* sp. (ZX22B) (Figure 2) and there are few base pair differences in ITS, *tef1-α*, and *tub2*. Morphologically, our new collections share similar conidial size and number of appendages with *Neopestalotiopsis* sp.2 (BJFC-S1790), although there were slight differences in conidial color. The median cell of strain CGMCC3.23553 (Figure 8) isolated from *Paeonia suffruticosa* has darker conidia than *Neopestalotiopsis* sp.2 from *Castanea mollissima* leaves. In contrast, another strain isolated from diseased olive leaves (UESTCC 22.0034) shares similar conidial color to *Neopestalotiopsis* sp.2 (BJFC-S1790) (Figure 9). Differences in the conidial color appear to vary depending on the condition of the substrates (herbaceous or woody plants). Therefore, we regard these specimens as conspecific and named *Neopestalotiopsis terricola*.

#### 3.2.4. *Pestalotiopsis kenyana* Maharachch., K.D. Hyde and Crous, Studies in Mycology, 79:121–186 (Figure 10 and Figure 11)

MycoBank: MB 809741

*Pathogenic* on diseased branches of *Camellia oleifera*. Sexual morph: *Ascomata* 262–298 μm diam., 235–253 μm high, solitary, gregarious, scattered, immersed under host epidermis, dark brown to black, subglobose, ostiolate with minute papillate black, periphysate. *Peridium* is 23–25 μm thick, with several layers of dark brown to hyaline pseudoparenchymatous cells, arranged in textura angularis. *Hamathecium* is 3–4.5 μm wide, and composed of numerous, cylindrical, septate, unbranched, hyaline, paraphyses. Asci 71–93 × 7–9.5 μm ($\bar{x}$ = 82 × 8.5 μm, *n* = 30), cylindrical to clavate, containing eight (obliquely) uniseriate ascospores, unitunicate, thin-walled, short pedicellate with knob-like pedicel, apically rounded. *Ascospores* 12.5–14.5 × 5–6 μm ($\bar{x}$ = 13.5 × 5.5 μm, *n* = 30) μm, ellipsoid, yellow to dark brown, ellipsoidal to fusiform with obtuse ends, 3-euseptate often thicker than the wall, straight to slightly curved, smooth-walled. Asexual morph: *Conidiomata* mostly solitary, blackish brown, immersed, or semi-erumpent. Conidiophores are mostly reduced to conidiogenous cells. The *Conidiogenous cells* are 2–4 × 2–2.5 μm ($\bar{x}$ = 3 × 2.5 μm, *n* = 30) discrete, subcylindrical or ampulliform, hyaline, smooth, thin-walled. *Conidia* 17.5–22 × 6–7 μm ($\bar{x}$ = 20 × 6.5 μm, *n* = 30), fusoid, ellipsoid, straight or slightly curved, 4-septate, basal cell obconic with a truncate base, hyaline, verruculose and thin-walled, 3–4 μm long; three median cells dolliform to subcylindrical, 14–15.5 μm long, concolorous, septa darker than rest of the cell (second cell from base 3.5–5 μm long; third cell 4–5 μm long; fourth cell 4–5.5 μm long); apical cell 2.5–3.5 μm long, hyaline, subcylindrical or obconical and thin-walled, with 3–4 tubular apical appendages, arising from the apical crest, unbranched, filiform, 7.5–11 μm long; basal appendage single, tubular, unbranched, centric, 3.5–5.5 μm long.

**Figure 10:**
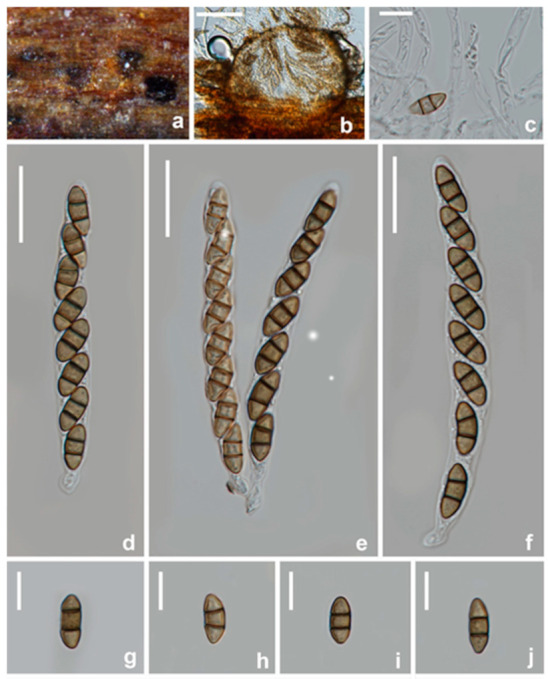
*Pestalotiopsis kenyana* (Sexual morph, HKAS 123214) (**a**) Appearance of ascomata on host substrate. (**b**) Section of ascoma. (**c**) paraphyses. (**d**–**f**) asci. (**g**–**j**) Ascospores. Scale bars: (**b**) = 100 μm, (**c**,**g**–**j**) = 10 μm, (**d**–**f**) = 20 μm.

**Figure 11:**
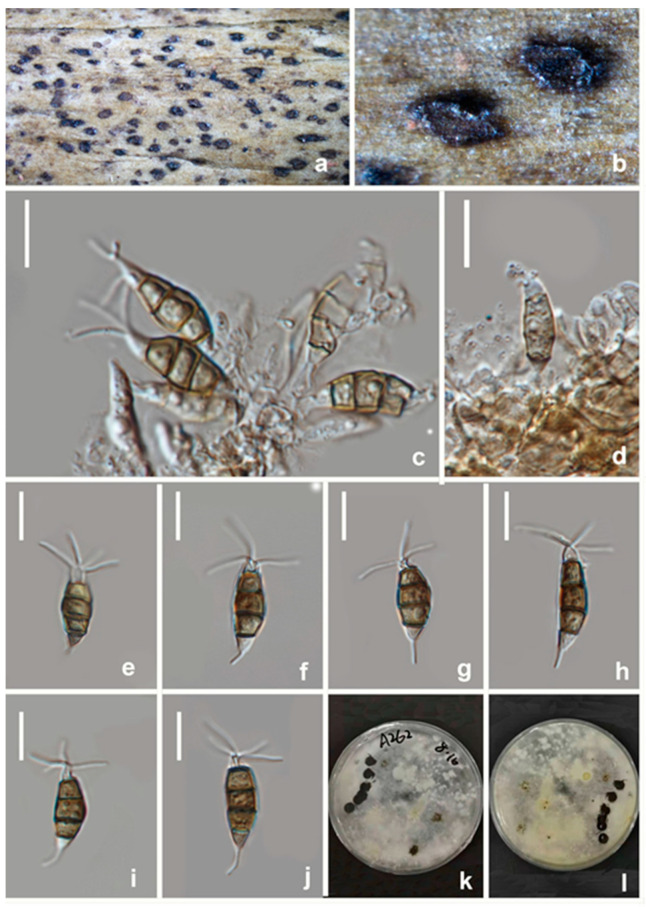
*Pestalotiopsis kenyana* (asexual morph, HKAS 123215) (**a**,**b**) Appearance of conidiomata on host substrate. (**c**,**d**) Conidiogenous cells and conidia. (**e**–**j**) Conidia. (**k**,**l**) Upper and reverse view of the colony on PDA. Scale bars: (**c**–**j**) = 10 μm.

Culture characteristics: Colonies on PDA reaching 60–70 mm diam. after 7 d at 25 °C, with smooth edge, whitish, with sparse aerial mycelium on the surface, reserve similar color.

Material examined: CHINA, Sichuan Province, Leshan city, on a diseased branch of *Camellia oleifera*, 23 June 2021, T. Zhang, YC A243 (HUEST 22.0038), living culture UESTCC 22.0037; *ibid*., 23 July 2021, Wenli Li, YC 348B (HKAS 123214, HUEST 22.0035); Mianyang city, on a diseased branched of *Paeonia suffruticosa*, 10 June 2021, W.L. Li, YMD A262 (HKAS 123215), living culture CGMCC3.23556. *ibid*., YMD A248 (HUEST 22.0039), living culture UESTCC 22.0038. *ibid*., YMD A255 (HUEST 22.0037), living culture UESTCC 22.0036. *ibid*., YMD 334 (HKAS 123216), living culture CGMCC3.23557.

Notes: Seven strains obtained in this study clustered with *Pestalotiopsis kenyana* (CBS 442.67 and LC 6633) with moderate bootstrap support (ML/BI 88%/0.99) (Figure 3). The comparison of ITS, *tef1-α*, and *tub2* genes showed there are only a few base pair differences. Furthermore, the strains CGMCC3.23556, UESTCC 22.0036, UESTCC 22.0037, UESTCC 22.0038, CGMCC 3.23557, and UESTCC 22.0035 show similar characteristics of asexual morph with *P. kenyana* (CBS 446.27) in conidial size and shape. Thus, we identified these newly obtained isolates as *P. kenyana*, and report this species from China for the first time. Specimens HKAS 123,214 (Figure 10) is the first sexual morph reported for *P. kenyana* based on molecular DNA data. Unfortunately, the conidia of HKAS 123,214 did not germinate in an artificial medium, and we were unable to observe its asexual morph from culture. In addition, *P. kenyana* (HKAS 123214) is morphologically similar to the sexual morph of *P. trachicarpicola*, as they share similar morphological characteristics in having immersed perithecia, ellipsoid and brown, 2-septate ascospores. However, *P. kenyana* differs from *P. trachicarpicola* as the latter has smaller ascomata (115–215 × 140–185 μm vs. 262–298 × 235–253 μm) and shorter asci (65–76 μm vs. 71–93 μm).

#### 3.2.5. *Seiridium ceratosporum* De Notaris, G. Memorie della Reale Accademia delle Scienze di Torino. Ser. 2. 3: 55–68 (1841) (Figure 12)

MycoBank: MB 359665

*Pathogenic* on diseased branches of *Vernicia fordii*. Sexual morph: not observed. Asexual morph: *Conidiomata* are sporodochial, mostly solitary, immersed to semi-erumpent, unilocular, conical. or subglobose with a flattened base, dark brown to black. *Conidiophores* are 34–55 × 2.5–3 μm ($\bar{x}$ = 40 × 2.5 μm, *n* = 30), septate, cylindrical, irregularly branched, hyaline or pale brown, thin-walled. *Conidiogenous cells* are 7–15 × 2–3.5 μm ($\bar{x}$ = 11 × 2.5 μm, *n* = 30), discrete, hyaline, cylindrical, smooth- and thin-walled, with moderate periclinal thickenings in collarette zone, colorless, with up to five proliferations. *Conidia* are 26–32 × 8.5–10.5 μm ($\bar{x}$ = 29 × 10 μm, *n* = 30), lunate to falcate, curved, 5-septate, rarely 4- or 6-septate, not striate, occasionally conidia formed in basipetal chains, bearing hyaline appendages at both ends, euseptate, basal cell obconic with a truncate base, hyaline to paler brown, walls smooth, 2.5–4 μm; four median cells, 18–24 μm long, smooth, cylindrical to doliiform, brown to dark brown, second cell from base 4.5–6 μm long, third cell 4–5.5 μm long, fourth cell 4.5–5.5 μm long, fifth cell 5–6.5 μm long, apical cell conical, hyaline to pale brown, smooth, 2–3.5 μm long, apical appendage single, cylindrical, mostly excentric, 4–6 μm long; basal appendage single, cylindrical, excentric, unbranched, 2.5–3.5 μm long.

**Figure 12:**
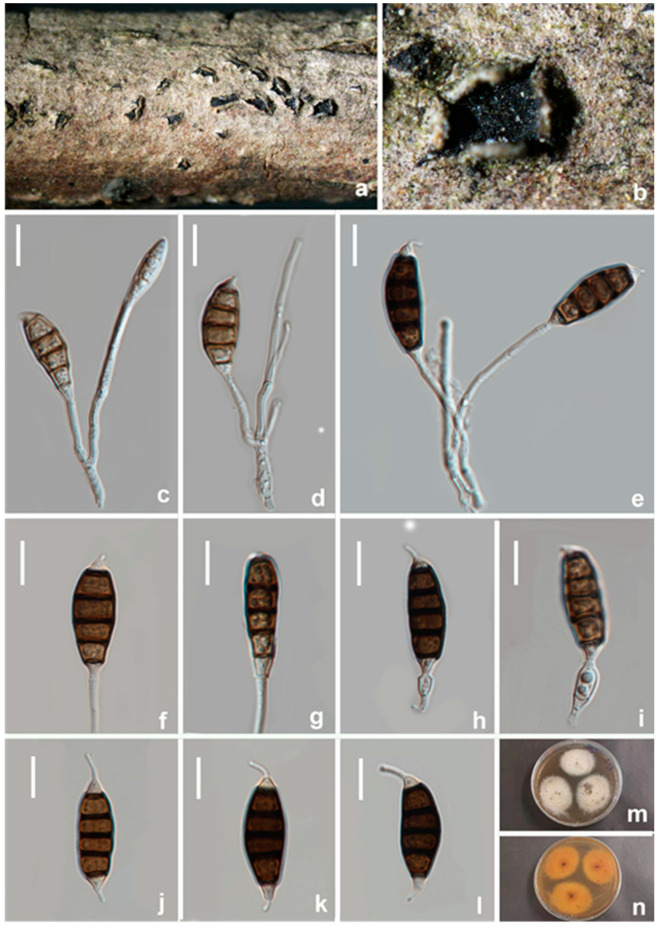
*Seiridium ceratosporum* (HKAS 123218) (**a**,**b**) Appearance of conidiomata on host substrate. (**c**–**e**) Conidiophores and conidia. (**f**–**i**) Conidiogenous cells and conidia. (**j**–**l**) Conidia. (**m**,**n**) Upper and reverse view of the colony on PDA. Scale bars: (**c**–**l**) = 10 μm.

Culture characteristics: Colonies on PDA reaching 20 mm diam. after 2 weeks at 25 °C, circular to irregular, medium dense, flat or effuse, with edge fimbriate, white to gray from above, light yellow to brown from below, producing pigments in agar.

Materials examined: CHINA, Sichuan Province, Guangyuan city, on a diseased branch of *Vernicia fordii*, 19 April 2021, T. Zhang, YT A106 (HKAS 123218), living culture, CGMCC3.23559; *ibid*., T. Zhang, YT A102 (HUEST 22.0040), living culture, UESTCC 22.0039; *ibid*., T. Zhang, YT A64 (HUEST 22.0041), living culture UESTCC 22.0040.

Notes: *Seiridium ceratosporum*, isolated from *Olea* sp. in Italy, was first described as *Stilbospora ceratospora* by De Notaris [40]. Nag Raj [41] examined the holotype material and transferred it to *Seiridium*. Though Liu et al. [42] provided the ITS and *tub2* for non-type strains of “*Seiridium ceratosporum* PHSI2001Pathcw07”, they did not provide any morphological data. In this study, three new strains obtained from *Vernicia fordii* clustered with “*Seiridium ceratosporum* PHSI2001Pathcw07” with high bootstrap support (100%). A comparison of sequence data between the strain PHSI2001Pathcw07 and our new strains shows a few nucleotides difference in ITS and *tub2*. Morphologically, the new collection (HKAS 123218) and ex-type strains of *Seiridium ceratosporum* share similar characteristics in the shape and size of conidia and conidiophores, while occasionally conidia formed in basipetal chains in our new collections. Thus, these three strains were identified as *S. ceratosporum* based on both molecular analyses and morphology. This is the first report of *S. ceratosporum* on the host *Vernicia fordii*.

#### 3.2.6. *Seiridium guangyuanum* W.L. Li and Jian K. Liu, sp. nov. (Figure 13)

MycoBank: MB 845409; Facesoffungi number: FoF 12749

Etymology: The name reflects the location Guangyuan city where the fungus was collected.

Holotype: HKAS 123219

*Pathogenic* on diseased branches of *Vernicia fordii*. Sexual morph: not observed. Asexual morph: *Conidiomata* is 106–108 μm high, 13–20 μm diam. ($\bar{x}$ = 107 × 12 μm, *n* = 15), sporodochial, scattered to gregarious, immersed to erumpent from tissue, initially closed and globose to subglobose, later dehiscing by a split in the overlying tissue and then appearing broadly conical in sectional view, unilocular, occasionally convoluted, glabrous black. The *Conidiomata wall* is 38–45 μm thick at the sides, not well defined, comprising brown, thin-walled cells of textura angularis, with lighter cells at the base fusing into the host tissue. *Conidiophores* septate, cylindrical, irregularly branched, branch lengths variable (22–42.5 μm long), occasionally reduced to conidiogenous cells, hyaline or paler brown, thin- and smooth-walled. *Conidiogenous cells* 11.5–16 × 2–2.5 μm ($\bar{x}$ = 14 × 2 μm, *n* = 30), discrete, hyaline, cylindrical to subcylindrical with moderate periclinal thickenings in the collarettes zone, smooth- and thin-walled, proliferating percurrently. *Conidia* are 27–30 × 8–9 μm ($\bar{x}$ = 28.5 × 8.5 μm, *n* = 30), lunate to falcate, straight or slightly curved, 5-septate, bearing appendages; basal cell obconical with a truncate base, hyaline, smooth-walled, 3–4 μm long; four median cells, 20–23 μm long, smooth, short cylindrical, thick-walled and smooth, septal pores distinctly visible, yellowish-brown to brown, and septa darker than the rest of the cells, second cell from base 5–6.5 μm long, third cell 5–6 μm long, fourth cell 4.5–5.5 μm long, fifth cell 4.8–6 μm long; apical cell conical, hyaline, thin-walled, smooth, 2.5–3.5 μm long, apical appendage single, mostly excentric, 2.5–5.5 μm long; basal appendage, when present, single, unbranched, centric, 3–4.5 μm long; mean conidium length/width ratio = 3.5:1.

**Figure 13:**
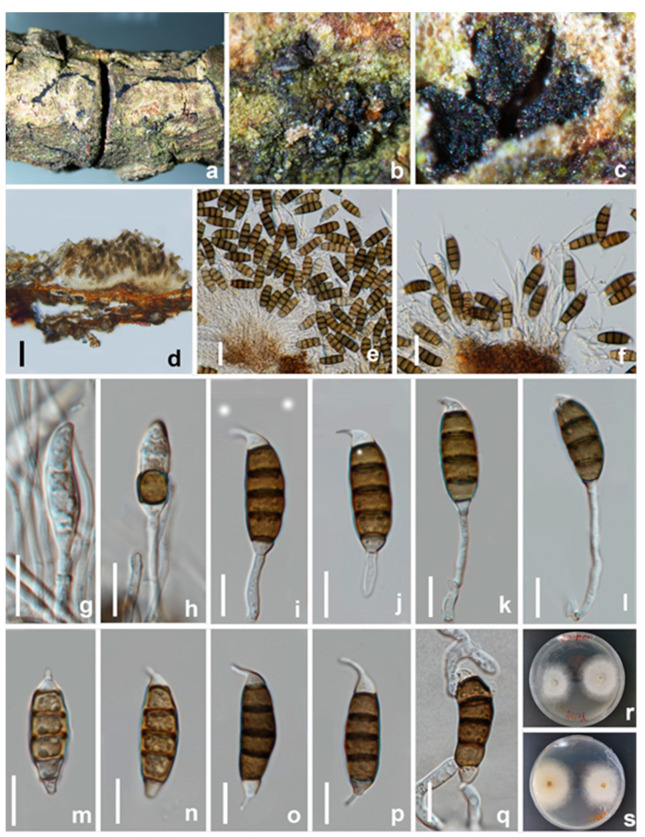
*Seiridium guangyuanum* (HKAS 123219, holotype) (**a–c**) Appearance of conidiomata on host substrate. (**d**) vertical section of conidiomata. (**e**,**f**) Sporodochia. (**g**–**l**) Conidiophore and conidia. (**m**–**p**) conidia. (**q**) Germinating conidium. (**r**,**s**) Upper and reverse view of the colony (on PDA). Scale bars: (**d**) = 50 μm, (**e**,**f**) = 20 μm, (**g**–**q**) = 10 μm.

Culture characteristics: Colonies on PDA reaching 20 mm diam. after 2 weeks at 25 °C, from above dense, aerial mycelium on a surface flat or raised, effuse with floccose texture, margin crenate and filamentous, white to cream at the margin, white in the centre, and reverse similar in colour.

Materials examined: CHINA, Sichuan Province, Guangyuan city, on a diseased branch of *Vernicia fordii*, 19 April 2021, W.L. Li, YT 184 (HKAS 123219, holotype), ex-type living culture, CGMCC3.23561; *ibid*., T. Zhang, YT 44 (HUEST 22.0042, isotype), ex-isotype living culture, UESTCC 22.0041; *ibid*., W.L. Li, YT200, (HUEST 22.0045), living culture, UESTCC 22.0044; *ibid*., W.L. Li, YT237, (HUEST 22.0048), living culture, UESTCC 22.0047; *ibid*., W.L. Li, YT243, (HUEST 22.0038), living culture, UESTCC 22.0037; Jiangyou city, on a diseased branch of *Camellia oleifera*, 10 June 2021, Z.P. Liu, YC29, (HUEST 22.0047), living culture, UESTCC 22.0046; *ibid*., YC 30, (HUEST 22.0046), living culture, UESTCC 22.0045. Chengdu city, on a diseased branch of *Olea europaea*, 20 January 2021, Z.P. Liu, GLL 17, (HUEST 22.0044), living culture, UESTCC 22.0043.

Notes: Eight strains of *Seiridium guangyuanum* formed a separate lineage in the muti-gene phylogeny as sister to *S. ceratosporum* (PHSI2001Pathcw07) with high bootstrap support (ML/BI 100%/1). *Seiridium guangyuanum* (CGMCC3.23561) is distinct from *S. ceratosporum* by 12 bp differences in ITS (508 bp, no gap) (Table 3) and 5 bp differences in *tub2* (750 bp, no gaps). Morphologically, *Seiridium guangyuanum* differs from *S. ceratosporum* by conidial (27–30 μm vs. 29–35 μm) and apical appendage measurements (2.5–5.5 μm vs. 4–8 μm), their mean conidium length/width ratio is distinct (3.5:1 vs. 2.9:1). Additionally, the colony of *S. ceratosporum* produces water-soluble brownish pigment in PDA. However, this trait was not observed in *Seiridium guangyuanum* under the same culture conditions.

#### 3.2.7. *Seiridium oleae* W.L. Li and Jian K. Liu, sp. nov. (Figure 14)

MycoBank: MB 845410; Facesoffungi number: FoF 12750

Etymology: The name reflects the host plant genus Olea, from which it was isolated.

Holotype: HKAS 123220

*Pathogenic* on diseased branches of *Olea europaea*. Sexual morph: not observed. Asexual morph: *Conidiomata* is sporodochial, mostly solitary, immersed to semi-erumpent, unilocular, conic, or subglobose with a flattened base, and dark brown to black. The *Conidiomata wall* is 24–27 μm thick at the sides, not well defined, comprising brown, thin-walled cells of *textura angularis*, with lighter cells at the base fusing into the host tissue. *Conidiophores* are up to 12 μm long, septate, cylindrical, irregularly branched, hyaline or paler brown, and thin-walled. *Conidiogenous cells* are 6–8.5 × 2.5–3 μm (=7 × 2.5 μm, *n* = 30), discrete, hyaline, cylindrical, smooth- and thin-walled, proliferating percurrently. *Conidia* is 20–26 × 7.5–9 μm (=23 × 8 μm, *n* = 30), lunate to falcate, curved, 5-septate, rarely 4- or 6-septate, not striate, bearing hyaline appendage at both ends, euseptate, basal cell obconic with a truncate base, hyaline to paler brown, walls smooth, 2.5–3.5 μm; four median cells, 19–22 μm long, smooth, cylindrical to doliiform, brown to dark brown, septa darker than the rest of the cells, second cell from base is 4–5.5 μm long, the third cell is 3.5–4.5 μm long, the fourth cell is 4–4.5 μm long, the fifth cell is 4–5 μm long, the apical cell is conical, hyaline to paler brown, smooth, 1.5–3 μm long, the apical appendage is single, cylindrical, mostly excentric, and 3–5 μm long; the basal appendage is single, cylindrical, excentric, unbranched, and 2.5–3.5 μm long, and the mean conidium length/width ratio = 2.8:1.

**Figure 14:**
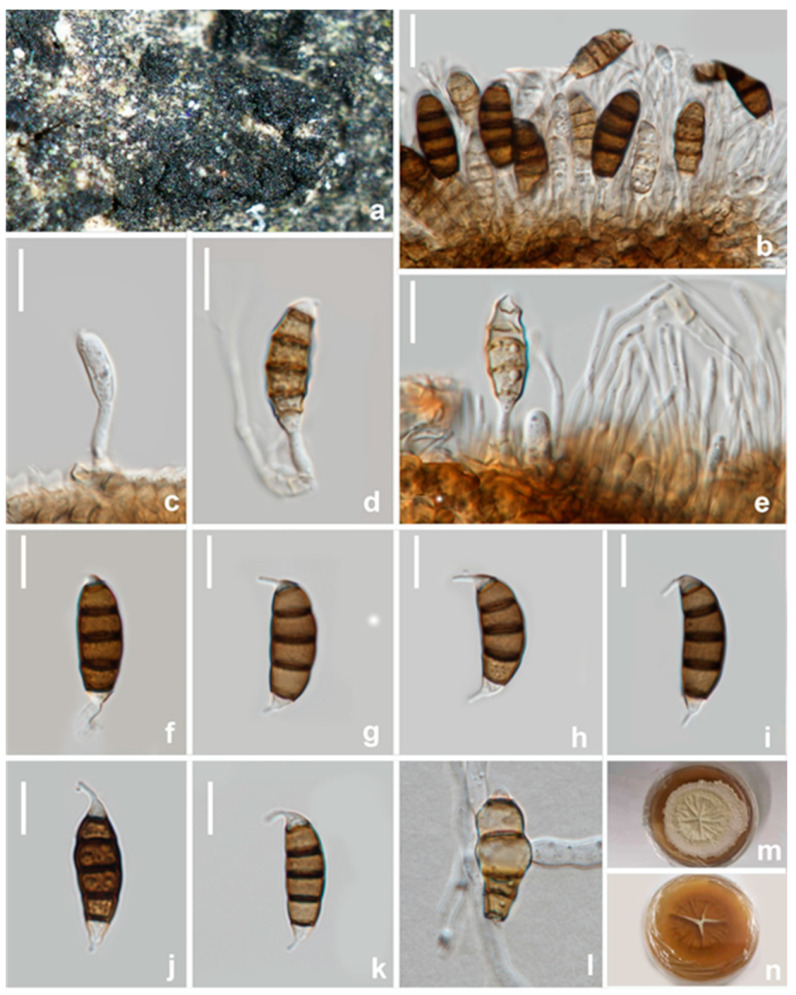
*Seiridium oleae* (HKAS 123220, holotype). (**a**) Appearance of conidiomata on host substrate. (**b**–**f**) Conidiogenous cells and conidia. (**g**–**k**) Conidia. (**l**) Germinating conidium. (**m**,**n**) Upper and reverse view of the colony on PDA. Scale bars: (**b**–**l**) = 10 μm.

Culture characteristics: Colonies on PDA reaching 20 mm diam. after 2 weeks at 25 °C are dense, circular, flattened to slightly raised, their surfaces are rough, radially furrowed at the centre, and smooth at the margin, with edge indented and velvety from above, whitish at the margin, pale yellowish at the centre, and from below, honey yellowish, producing yellowish pigmentation on agar medium.

Materials examined: CHINA, Sichuan Province, Guangyuan city, on a diseased branch of *Olea europaea*, 20 April 2021, T. Zhang, GL A94 (HKAS 123220, holotype), ex-type living culture, CGMCC3.23558; *ibid*., HUEST 22.0052, isotype, ex-isotype living culture, UESTCC 22.0051.

Notes: In the phylogenetic analysis, *Seiridium oleae* (CGMCC3.23558 and UESTCC 22.0048) groups with *S. rosarum* (CBS 442.67) with high bootstrap support (ML/BI 98%/0.99). These two species share similar morphological characters in conidia shape and dimension. However, *S. rosarum* (CGMCC3.23562) differs from *S. oleae* in having a longer apical appendage (up to 12 μm vs. 3–5 μm). Additionally, *Seiridium oleae* is separated from *S. rosarum* (CGMCC3.23562) by 10 bp differences in ITS (526 bp), 4 bp differences in LSU (818 bp), 18 bp differences in *rpb2* (900 bp), 46 bp differences in *tef1-α*, and 28 bp differences in *tub2*.

#### 3.2.8. *Seiridium rosarum* Wanas., Camporesi, E.B.G. Jones and K.D. Hyde, Fungal Diversity 89: 199 (2018) (Figure 15)

MycoBank: MB 554225

*Pathogenic* on diseased branches of *Paeonia suffruticosa*. Sexual morph: not observed. Asexual morph: *Conidiomata* are 116–156.5 μm high, 250–288 μm diam. ($\bar{x}$ = 136 × 269 μm, *n* = 15), pycnidial to sporodochial, mostly solitary, immersed to semi-erumpent, unilocular, conical, or subglobose with a flattened base, and dark brown to black. The *Conidiomata wall* is 30–36 μm thick at the sides, not well defined, comprising brown, thin-walled cells of *textura angularis*, with lighter cells at the base fusing into the host tissue. *Conidiophores* are septate, cylindrical, irregularly branched, with branch lengths variable (up to 40 μm long), hyaline or paler brown, thin-and smooth-walled. *Conidiogenous cells* are 7–12 × 2–3 μm ($\bar{x}$ = 9.5 × 2.5 μm, *n* = 30), discrete, hyaline, cylindrical, and proliferating percurrently. *Conidia* are 24–28 × 8.5–10 μm ($\bar{x}$ = 26 × 9 μm, *n* = 30), lunate to falcate, curved, 5-septate, not striate, bearing a hyaline appendage at both ends, euseptate, basal cell obconic with a truncate base, hyaline to paler brown, 3–4.5 μm long; four median cells, 19–23 μm long, verruculose, cylindrical to doliiform, and brown to dark brown, with septa darker than the rest of the cells, the second cell from base is 5.5–7 μm long, the third cell is 5–6 μm long, the fourth cell is 4.5–6 μm long, and the fifth cell is 5–6 μm long, the apical cell is conical, with hyaline to pale brown, and a smooth, 2.5–4 μm long, apical appendage mostly single, centric, and 2.5–5.5 μm long, occasionally branched; the basal appendage is single, cylindrical, excentric, unbranched, and 3–4.5 μm long, with a mean conidium length/width ratio = 2.8:1.

**Figure 15:**
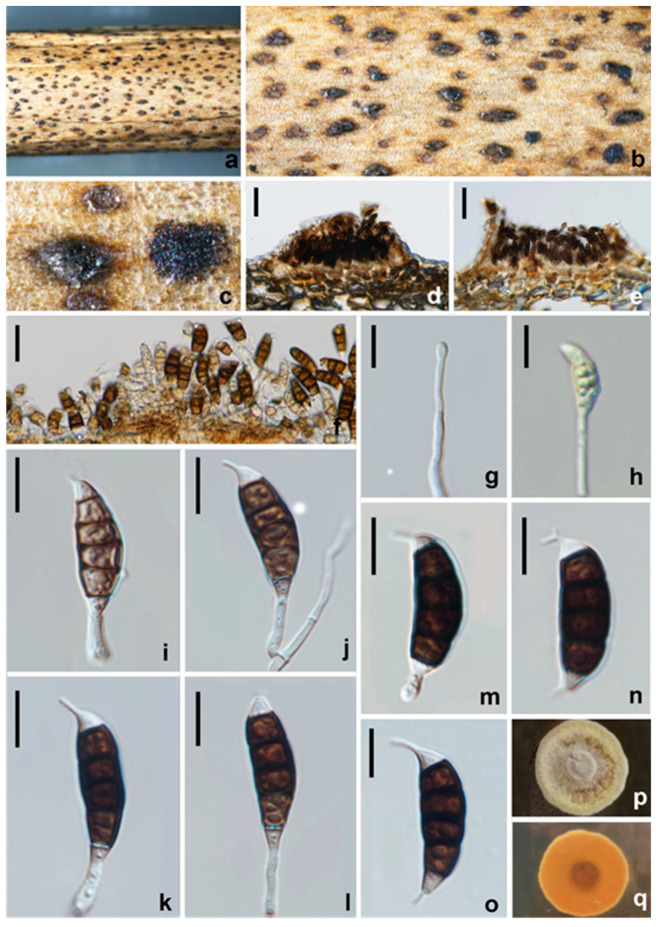
*Seiridium rosarum* (HKAS 123217). (**a**–**c**) Appearance of conidiomata on host substrate. (**d**,**e**) Vertical section of conidiomata. (**f**–**l**) Conidiogenous cells and conidia. (**m**–**o**) Conidia. (**p**,**q**) Upper and reverse view of the colony on PDA. Scale bars: (**d**,**e**) = 50 μm, (**f**) = 20 μm, (**g**–**o**) = 10 μm.

Culture characteristics: Colonies on PDA reaching 20 mm diam. after 2 weeks at 25 °C. Cultures from above are paler-yellowish, dense, circular, umbonate, papillate with fluffy, covered with white aerial mycelium; reverse orange, paler-yellowish at the edge.

Material examined: CHINA, Sichuan Province, Leshan city, on a diseased branch of *Paeonia suffruticosa*, 23 July 2021, W.L. Li, YMD 328 (HKAS 123217), living culture, CGMCC3.23562; Miangyang city, on a diseased branch of *Paeonia suffruticosa*, 10 September 2021, W.L. Li, YMD 483 (HUEST 22.0049), living culture, UESTCC 22.0048.

Notes: Phylogenetic analyses indicated two isolates from the *Paeonia suffruticosa* cluster with *S. rosarum* (MFLUCC 17–0654), with high bootstrap support (ML/BI 98%/1.00). *Seiridium rosarum* was isolated from *Rosa canina* in Italy, and only LSU and ITS sequences are available for this species. There are seven bp differences across 537 bp in ITS, zero bp differences across 815 bp in LSU between *S. rosarum*, and two isolates obtained in this study. Morphologically, these two isolates are similar to *S. rosarum* in having similar conidial dimensions (Table 4) and verruculose, concolorous median cells. However, our isolates show appendages at both ends of conidia and apical appendages branched occasionally. Based on morphology and DNA data, we identified our collections as *Seiridium rosarum* and report it as a new record in China.

#### 3.2.9. *Seiridium vernicola* W.L. Li and Jian K. Liu, sp. nov. (Figure 16)

MycoBank: MB 845411; Facesoffungi number: FoF 12751

Etymology: The name reflects the host plant genus Vernicia, from which it was isolated.

Holotype: HKAS 123221

*Pathogenic* on diseased branches of *Vernicia fordii*. Sexual morph: not observed. Asexual morph: *Conidiomata* are 145–192 μm high, 279–416 μm diam. ($\bar{x}$ = 169 × 347 μm, *n* = 15), sporodochial, mostly solitary, immersed to erumpent from tissue, unilocular, conical, or cupulate with a flattened base, and black. The *Conidiomata wall* is 48–64.5 μm thick at the sides, not well defined, comprising brown, thin-walled cells of textura angularis, with lighter cells at the base fusing into the host tissue. *Conidiophores* are septate, cylindrical, irregularly branched, with variable branch lengths (33–64 μm long), hyaline or pale brown, and thin-and smooth-walled. *Conidiogenous cells* are 5.5–10 × 2–3 μm (=8 × 2.5 μm, *n* = 30), discrete, hyaline, cylindrical, smooth- and thin-walled. *Conidia* is 25–32 × 8–10 μm (=28 × 9 μm, *n* = 30), lunate to falcate, curved, 5-septate, rarely 4- or 6-septate, not striate, bearing one appendage, basal cell obconic with a truncate base, hyaline, smooth-walled, and 4–5 μm long, with four median cells, 18–25 μm long, smooth, cylindrical to doliiform, brown to dark brown, with septa darker than the rest of the cells; the second cell is from base 4–5 μm long, the third cell is 4.5–6.5 μm long, the fourth cell is 4–5.5 μm long, the fifth cell is 4.5–6.5 μm long, and the apical cell is conical, hyaline, smooth, 3–4.5 μm long, with a single apical appendage, mostly excentric, 2.5–5 μm long, with a basal appendage of 1–1.5 μm long, and a mean conidium length/width ratio = 3:1.

**Figure 16:**
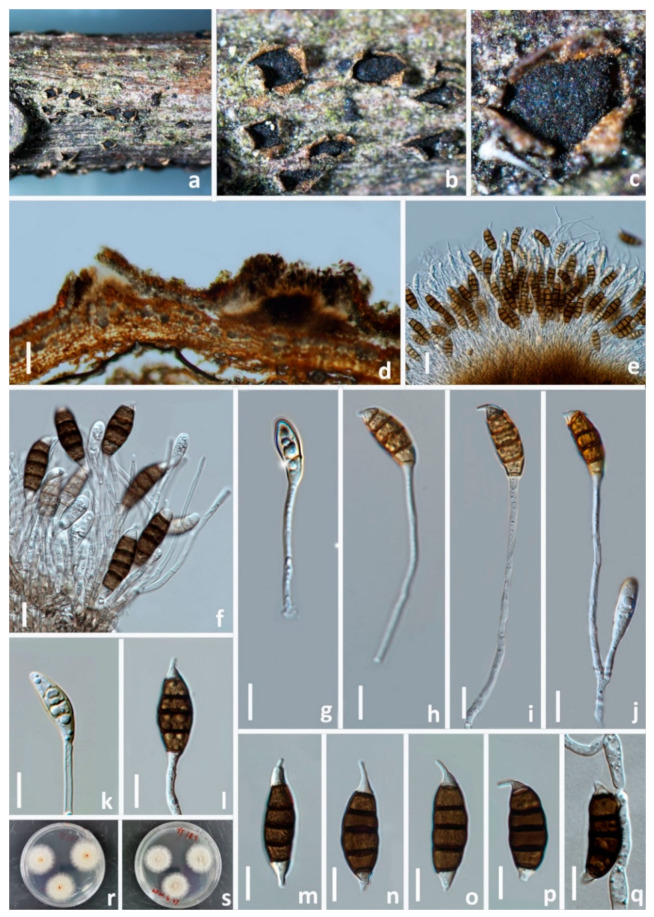
*Seiridium vernicola* (HKAS 123221, holotype). (**a**–**c**) Appearance of conidiomata on the host. (**d**) vertical section of conidiomata. (**e**,**f**) Sporodochia. (**g**–**j**) Conidiophores and conidia. (**k**,**l**) Conidiogenous cells and conidia. (**m**–**p**) Conidia. (**q**) Germinating conidium. (**r**,**s**) Upper and reverse view of the colony on PDA. Scale bars: (**d**) = 100 μm, (**e**) = 20 μm, (**f–q**) = 10 μm.

Culture characteristics: Colonies on PDA reaching 20 mm diam. after 2 weeks at 25 °C, from above, cream with white, dense, circular, umbonate, papillate with fluffy, covered with white aerial mycelium.

Material examined: CHINA, Sichuan Province, Guangyuan City, on a diseased branch of *Vernicia fordii*, 19 April 2021, W.L. Li, YT 183 (HKAS 123221, holotype), ex-type living culture, CGMCC3.23560; Chengdu city, on a diseased branch of *Sapium sebiferum*, 5 March 2021, T. Zhang, A6 (HUEST 22.0050), living culture UESTCC 22.0049; *ibid*., A10 (HUEST 22.0051), living culture UESTCC 22.0050.

Notes: Three strains of *Seiridium vernicola* formed a distinct clade in the multi-locus phylogenetic tree. *Seiridium vernicola* is morphologically similar to the species of *Seiridium* (*S. marginatum*) and *S. venetum* in having hyaline, cylindrical, and relatively long conidiophores. However, *S. marginatum* differs from *S. vernicola* in having larger conidia (38–42 μm vs. 25–32 μm) and well-developed appendages (apical appendage: 3.5–7.5 μm vs. 2.5–5 μm; basal appendage: 1.5–5.5 vs. 1–1.5 μm). In addition, the conidia of *S. marginatum* have striate surfaces, a feature not observed in *S. vernicola*. *Seiridium venetum* differs from *S. vernicola* in having narrower conidia (6.5–8.5 μm vs. 8–10 μm) with branched appendages.

### 3.3. Pathogenicity Assay

**Figure 17:**
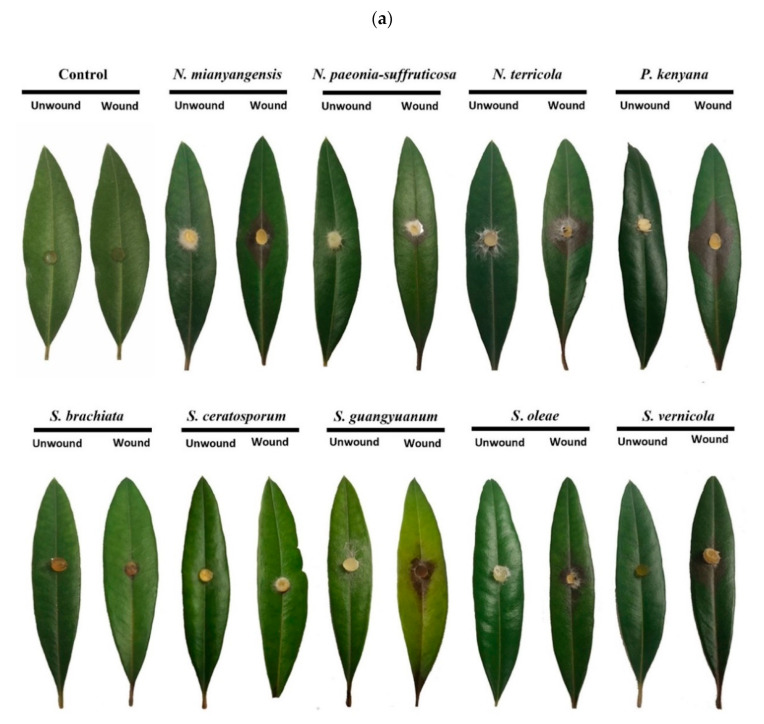

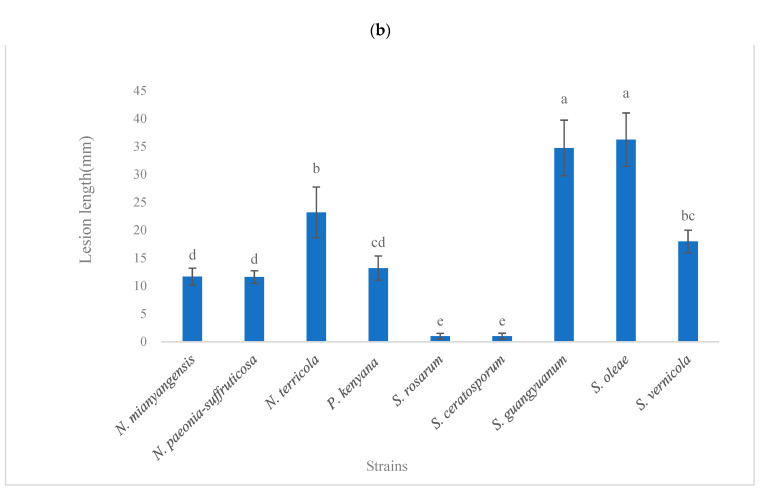
Pathogenicity test results of nine pestalotiod species on olive leaves. (**a**) Induced symptoms on wounded and unwounded olive leaves after 7 d. (**b**) Virulence of the isolates was evaluated by measuring lengths of the necrotic lesions in millimeters on infected olive leaves after 7 d. Error bars indicate the standard deviation of the mean. Significant differences (*p* < 0.05) between means are indicated with different letters according to Duncan’s multiple range test.

Results of pathogenicity tests were determined 7 d after inoculation. Seven species out of nine produced brown lesions showing virulence on wounded leaves. The isolates of *Seiridium oleae* were the most aggressive, causing significantly longer f brown lesions (average lesion length 41 mm, SD = 8.5), followed by *S. guangyuanum* (average lesion length 35 mm, SD = 5) (Figure 17b). Both *Neopestalotiopsis* and *Pestalotiopsis* species displayed average size lesions, while *Seiridium brachiata* and *S. ceratosporum* did not show significant pathogenicity on olive leaves. No symptoms were observed on leaves using the unwounded method (Figure 17a) nor in any control group. Koch’s postulates were fulfilled by reisolating the same fungi and the colony and morphological characters were verified.

## 4. Discussion

Pestalotiod species have been extensively investigated on several woody oil plants globally, especially in Italy, Spain, and the USA [43,44,45], but no extensive studies have been carried out in China. We conducted wide-ranging surveys of pestalotiod fungi associated with diseased branches and leaves of commonly grown woody oil plants in Sichuan Province, resulting in 29 isolates. Based on multi-locus phylogeny and morphological analyses, six novel species (*Neopestalotiopsis mianyangensis*, *N. paeonia-suffruticosa*, *N. terricola*, *Seiridium guangyuan*, *S. vernicola*, and *S. oleae*) and three new records (*Pestalotiopsis kenyana*, *Seiridium ceratosporum*, and *S. rosarum*) were identified and described. Additionally, the sexual morph of *Pestalotiopsis kenyana* was reported for the first time in this study.

Conidial characters such as length, width, median cell length, the color of median cells, and length of the apical appendages are widely used for taxonomic purposes and inter-specific delineation of pestalotiod fungi. However, Maharachchikumbura et al. observed that some *Pestalotiopsis* species have similar conidial dimensions which cluster in distinct clades [13]. In this study, we observed that the conidial shape, size, and color of *Neopestalotiopsis terricola* isolated from *Paeonia suffruticosa* varied after inoculation on the olive leaves (Figure 7 and Figure 8). We found that the conidia of *N. paeonia-suffruticosa* on PDA are shorter than those naturally found on woody plants (Figure 7). Hence, we suggest that conidial length and width, even color, may not be a reliable taxonomic character alone for distinguishing *Neopestalotiopsis* species. Though the conidial appendages appear to be highly informative at the species level, the apical appendages vary in length, number, shape, branched or unbranched nature, presence or absence of knobbed tips, and the position of attachment to the conidial body [46]. Bonthond et al. [21] compared the appendages of most species in the genus *Seiridium* and found that many species have significant differences in the basal appendages. The same result was confirmed by our study.

Since pestalotiod species may have an endophytic, saprobic, or pathogenic lifestyle, we determined their pathogenicity on detached leaves of 3-year-old *Olea europaea* by inoculating colonized mycelial discs via two different methods (‘wounded’ and ‘unwounded’). The results showed that all tested species were unable to provoke brown lesions on detached and unwounded olive leaves; however, most of the pestalotiod fungi were able to infect the host in the wounded method, revealing that injuries to woody oil plants could lead to fungal infection. Moreover, these isolates showed different virulence spectra with *Seiridium oleae* isolates being highly aggressive to detached olive leaves, whereas *S. ceratosporum* was much less aggressive on the same olive leaves. These results showed that pestalotiod fungi are pathogenic and responsible for causing diseases of commonly grown woody oil plants by fulfilling Koch’s postulates.

It is worth noting that most pestalotiod species have a wide host range, and they have been reported to infect many economic plants resulting in severe diseases, e.g., avocado, blueberry [47], mango [48], strawberry [49], and kiwifruit [50]. As most pestalotiod species identified in this study were pathogenic to detached leaves of *Olea europaea*, they may pose potential threats to olive production by causing plant losses and reducing plant quality. The pathogenicity tests and host range studies indicated that pestalotiod fungi obtained from this study are not host-specific, and they can infect woody oil plants other than those from which they were initially isolated. This indicates that some *Neopestalotiopsis*, *Pestalotiopsis*, and *Seiridium* species can potentially infect a range of woody oil plants. The isolates were collected from different locations in the Sichuan Province, confirming the widespread distribution of the pathogen. Several biotic and abiotic factors, such as pest insects, hail, and many others, may cause injury to woody oil plants and could lead to fungal infection. To reduce the occurrence of diseases, the prevention of trunk wounds and the pruning of dead, dying, or diseased branches are likely to aid in reducing the incidence of disease.

Since woody oil plants are regarded as economical and commercial plant species in China, more extensive research on fungal pathogenic species from fresh material should be conducted to help clarify the pathogens and to verify the nature of the infection. This would contribute to potential intervention by taking preventive measures to reduce infection.

## Figures and Tables

**Table 1:** Isolates and GenBank accession numbers of sequences obtained in this study.

Species	Culture Accession No	Host/Substrate		GenBank Accession			
			LSU	ITS	*rpb2*	*tef1-α*	*tub2*
*Neopestalotiopsis mianyangensis*	UESTCC 22.0006	*Paeonia suffruticosa*	N/A	OP082291	N/A	OP204793	OP235979
*Neopestalotiopsis mianyangensis*	CGMCC 3.23554 *	*Paeonia suffruticosa*	N/A	OP546681	N/A	OP723490	OP672161
*N. paeonia-suffruticosa*	CGMCC 3.23555 *	*Paeonia suffruticosa*	N/A	OP082292	N/A	OP204794	OP235980
*N. paeonia-suffruticosa*	UESTCC 22.0033	*Paeonia suffruticosa*	N/A	OP082293	N/A	OP204795	OP235981
*N. terricola*	CGMCC 3.23553 *	*Paeonia suffruticosa*	N/A	OP082294	N/A	OP204796	OP235982
*N. terricola*	UESTCC 22.0034	*Paeonia suffruticosa*	N/A	OP082295	N/A	OP204797	OP235983
*Pestalotiopsis kenyana*	CGMCC 3.23557	*Olea europaea*	N/A	OP082296	N/A	OP204798	OP235984
*P. kenyana*	UESTCC 22.0035	*Olea europaea*	N/A	OP082297	N/A	OP204799	OP235985
*P. kenyana*	HUEST 22.0035	*Paeonia suffruticosa*	N/A	OP729296	N/A	OP204801	OP235987
*P. kenyana*	HKAS 123214	*Camellia oleifera*	N/A	OP082298	N/A	OP204800	OP235986
*P. kenyana*	CGMCC 3.23556	*Camellia oleifera*	N/A	OP082299	N/A	OP204802	OP235988
*P. kenyana*	UESTCC 22.0036	*Paeonia suffruticosa*	N/A	OP082301	N/A	OP204805	OP235991
*P. kenyana*	UESTCC 22.0037	*Paeonia suffruticosa*	N/A	OP082300	N/A	OP204803	OP235989
*Seiridium ceratosporum*	CGMCC 3.23559	*Vernicia fordii*	OP082278	OP082304	OP204825	OP204808	OP235994
*S. ceratosporum*	UESTCC 22.0039	*Vernicia fordii*	OP082277	OP082303	OP204824	OP204807	OP235993
*S. ceratosporum*	UESTCC 22.0040	*Vernicia fordii*	OP082276	OP082302	OP723486	OP204806	OP235992
*S. guangyuanum*	CGMCC 3.23561 *	*Vernicia fordii*	OP082279	OP082305	OP204826	OP204809	OP235995
*S. guangyuanum*	UESTCC 22.0041	*Vernicia fordii*	OP082280	OP082306	OP204827	OP204810	OP235996
*S. guangyuanum*	UESTCC 22.0042	*Vernicia fordii*	OP082281	OP082307	OP204828	OP204811	OP235997
*S. guangyuanum*	UESTCC 22.0043	*Olea europaea*	OP082282	OP082308	OP204829	OP204812	OP235998
*S. guangyuanum*	UESTCC 22.0044	*Vernicia fordii*	OP082283	OP082309	OP204830	OP204813	OP235999
*S. guangyuanum*	UESTCC 22.0045	*Camellia oleifera*	OP714449	OP082310	OP723487	OP204814	OP236000
*S. guangyuanum*	UESTCC 22.0046	*Camellia oleifera*	OP714450	OP082311	OP204831	OP204815	OP236001
*S. guangyuanum*	UESTCC 22.0047	*Vernicia fordii*	OP082284	OP082312	OP204832	OP204816	OP236002
*S. oleae*	CGMCC 3.23558 *	*Olea europaea*	OP082285	OP082313	OP204833	OP204817	OP236003
*S. oleae*	UESTCC 22.0051	*Olea europaea*	OP082286	OP082314	OP204834	OP204818	OP723489
*S. rosarum*	CGMCC 3.23562	*Paeonia suffruticosa*	OP082287	OP082315	OP204835	OP204819	OP236004
*S. rosarum*	UESTCC 22.0048	*Paeonia suffruticosa*	OP714451	OP082316	OP723488	OP204820	OP236005
*S. vernicola*	UESTCC 22.0049	*Sapium sebiferum*	OP082288	OP082317	OP204836	OP204821	OP236006
*S. vernicola*	UESTCC 22.0050	*Sapium sebiferum*	OP082289	OP082318	OP204837	OP204822	OP236007
*S. vernicola*	CGMCC 3.23560 *	*Vernicia fordii*	OP082290	OP082319	OP204838	OP204823	OP236008

Ex-type strains are indicated with *; “N/A” denotes sequences that are not available.

**Table 2:** Comparison of the conidial dimension of *Neopestalotiopsis* species related to this study.

Species	Isolate Number	Conidial Size (μm)	Apical Appendages (μm)		Basal Appendage
			Number	Length	
*Neopestalotiopsis chiangmaiensis*	MFLUCC 18-0113	18–22 × 8–11	(2–)3	4–28	3–5
*N. cubana*	CBS 600.96	(19–)20–25(–27) × (7.5–)8–9.5(–10)	2–4	(19–)21–27(–28)	4–7
*N. dendrobii*	MFLUCC 14-	(19–)20.5–23(–24.5) × (6–)6.5–7.5 (–8)	2–3(2)	(4–)5–6.5(–6.6)	NA
*N. mianyangensis*	UESTCC 22.0006	19–23 × 5.5–7	3	5.5–11	3–4
*N. paeonia-suffruticosa*	CGMCC3.23554	20–23 × 9–11	3–4	22.5–34	3.5–7.5
*N. pandanicola*	KUMCC 17-0175	27–35 × 7.5–11	2(–3)	9.5–26	3–6
*N. saprophyta*	MFLUCC 12-0282	22–30 × 5–6	2–4(3)	4–5	4–7

**Table 3:** Nucleotide variations in the ITS regions of the strains of *Seiridium ceratosporum* and the strains of *S. guangyuanum*.

Isolates	ITS											
	54	59	60	63	73	76	77	90	108	112	355	430
*Seiridium ceratosporum* PHSI2001Pathcw07	G	A	C	T	T	G	G	A	T	C	A	T
*S. ceratosporum* UESTCC 22.0040	G	A	C	T	T	G	G	A	T	C	A	T
*S. ceratosporum* UESTCC 22.0039	–	A	C	T	T	G	G	A	T	C	A	T
*S. ceratosporum* CGMCC3.23559	G	A	C	T	T	G	G	A	T	C	A	T
*S. guangyuan* UESTCC 22.0043	T	C	G	C	G	A	C	G	C	A	G	C
*S. guangyuan* CGMCC3.23561	T	C	G	C	G	A	C	G	C	A	G	C
*S. guangyuan* UESTCC 22.0044	T	C	G	C	G	A	C	G	C	A	G	C
*S. guangyuan* UESTCC 22.0047	T	C	G	C	G	A	C	G	C	A	G	C
*S. guangyuan* UESTCC 22.0037	T	C	G	C	G	A	C	G	C	A	G	C
*S. guangyuan* UESTCC 22.0046	T	C	G	C	G	A	C	G	C	A	G	C
*S. guangyuan* UESTCC 22.0045	T	C	G	C	G	A	C	G	C	A	G	C
*S. guangyuan* UESTCC 22.0041	T	C	G	C	G	A	C	G	C	A	G	C

**Table 4:** Comparison of conidial dimension of *Seiridium* species related to this study.

Species	Isolated Number	Conidial Size	Apical Appendages (μm)		Basal Appendage
			Number	Length	
*Seiridium aquaticum*	MFLUCC 17-0474	29–35 × 12–14	NA	NA	NA
*S. ceratosporum*	Unknown	29–35 × 10–12(–12.5)	2	4–8(–11)	1–5(–6)
*S. ceratosporum*	CGMCC3.23559	26–32 × 8.5–10.5	2	4–6	2.5–3.5
*S. chinense*	CFCC 53031	(24–)25.5–28(–29.5) × (8–)8.5–9.5(–11)	2	4.5–15	6–18
*S. guangyuanum*	CGMCC3.23561	27–30 × 8–9	2	2.5–5.5	3–4.5
*S. marginatum*	CBS 140403	32–42(–47) × 7–9.5	2	32–52	22–44
*S. oleae*	CGMCC3.23558	20–26 × 7.5–9	2	3–5	2.5–3.5
*S. papillatum*	CBS 340.97	26.5–34.5 × 10–15	2	1.25–2.5	2
*S. rosarum*	MFLUCC 17-0654	22–28 × 7–9	1	3.5–4	up to 12
*S. rosarum*	CGMCC3.23562	24–28 × 8.5–10	2	2.5–5	3–4.5
*S. venetum*	MFLU 14-0265	20–30 × 6.5–8.5	2	10–35	2–5
*S. vernicola*	CGMCC3.23560	25–32 × 8–10	2	3–4.5	2.5–5

## Data Availability

The sequences data were submitted to GenBank.

## Supplementary Materials

The following are available online at https://www.mdpi.com/article/10.3390/jof8111175/s1: Table S1: Information of the genera *Neopestalotiopsis*, *Pestalotiopsis*, *Seiridium* isolates selected for phylogenetic analyses.
